# A Comprehensive Update of Various Attempts by Medicinal Chemists to Combat COVID-19 through Natural Products

**DOI:** 10.3390/molecules28124860

**Published:** 2023-06-20

**Authors:** Ayesha Rafiq, Tooba Jabeen, Sana Aslam, Matloob Ahmad, Usman Ali Ashfaq, Noor ul Amin Mohsin, Magdi E. A. Zaki, Sami A. Al-Hussain

**Affiliations:** 1 Department of Chemistry, Government College University, Faisalabad 38000, Pakistan; 2 Department of Chemistry, Government College Women University, Faisalabad 38000, Pakistan; 3 Department of Bioinformatics and Biotechnology, Government College University, Faisalabad 38000, Pakistan; 4 Department of Pharmaceutical Chemistry, Faculty of Pharmaceutical Sciences, Government College University, Faisalabad 38000, Pakistan; 5 Department of Chemistry, Faculty of Science, Imam Mohammad Ibn Saud Islamic University (IMSIU), Riyadh 11623, Saudi Arabia

**Keywords:** COVID-19, SARS-CoV-2, natural products, in vivo, in vitro, in silico, molecular docking, molecular docking simulations

## Abstract

The ongoing COVID-19 pandemic has resulted in a global panic because of its continual evolution and recurring spikes. This serious malignancy is caused by the severe acute respiratory syndrome coronavirus 2 (SARS-CoV-2). Since the outbreak, millions of people have been affected from December 2019 till now, which has led to a great surge in finding treatments. Despite trying to handle the pandemic with the repurposing of some drugs, such as chloroquine, hydroxychloroquine, remdesivir, lopinavir, ivermectin, etc., against COVID-19, the SARS-CoV-2 virus continues its out-of-control spread. There is a dire need to identify a new regimen of natural products to combat the deadly viral disease. This article deals with the literature reports to date of natural products showing inhibitory activity towards SARS-CoV-2 through different approaches, such as in vivo, in vitro, and in silico studies. Natural compounds targeting the proteins of SARS-CoV-2—the main protease (M^pro^), papain-like protease (PL^pro^), spike proteins, RNA-dependent RNA polymerase (RdRp), endoribonuclease, exoribonuclease, helicase, nucleocapsid, methyltransferase, adeno diphosphate (ADP) phosphatase, other nonstructural proteins, and envelope proteins—were extracted mainly from plants, and some were isolated from bacteria, algae, fungi, and a few marine organisms.

## 1. Introduction

The “Coronavirus Disease 2019” (COVID-19) was first found as an endemic in Wuhan, China, and was declared a global pandemic by the World Health Organization (WHO) on 11 March 2020 [1,2]. COVID-19 has become a persistent threat to public health and an international concern in the scientific community because of its rapid spread. To date (7 January 2023), more than 667 million cases and 6.70 million associated deaths of the coronavirus disease 2019 (COVID-19) have been recorded worldwide (https://www.worldometers.info/coronavirus/ (accessed on 7 January 2023)). COVID-19 is caused by “Severe Acute Respiratory Syndrome Coronavirus 2” (SARS-CoV-2), which is more infectious and fatal, with a reproductive rate of 2.5 (range 1.8–3.6) compared to the hemaglutinin1-neuraminidase1 (H1N1) influenza A virus, the SARS-CoV reproductive rate, i.e., (range 2–3), and the Middle East respiratory syndrome coronavirus (MERS-CoV) with a 0.9 reproductive rate [3]. Due to its rapid transmission from person to person, it has been considered a highly contagious malignancy, leading from a common cold or mild flu to a fatal disease [4]. Its common symptoms include a throat infection, headache, fever, fatigue, dry cough, breath shortness, body aches, loss of taste and smell, and pneumonia [5]. The symptoms become more severe if the affected person is elderly, has a weak immune system, or has suffered from other illnesses, such as obesity, diabetes, and cardiovascular and pulmonary disorders [6,7].

The infection of SARS-CoV-2 starts with the entry of the virion through interaction with the human ACE2 (angiotensin-converting enzyme 2) receptor cells using spike glycoproteins present on its surface [8]. Open reading frames (ORF1a and ORF1b) use host cell ribosomes to generate polyproteins pp1a and pp1b, which are then processed by main proteases to generate 16 nonstructural proteins (NSPs). All these NSPs play their own significant roles in the replication and transcription of the viral genome [9]. After synthesizing structural proteins, viral envelope formation is carried out in the endoplasmic reticulum–Golgi intermediate complex and released from the host cell through budding [10,11].

Coronaviruses are categorized into four classes: Alpha, Beta, Gamma, and Delta. MERS-CoV, SARS-CoV, and SARS-CoV-2 belong to the Beta class of the *Coronoviridae* family [12]. The novel SARS-CoV-2 is an enveloped, positive-sense single-stranded RNA virus with a genome length in the range of 80–120 nm [13]. It is reported that, genetically, the virus possesses similarities with SARS-CoV or MERS-CoV [14]. Studies have shown that the genetic material encodes four structural proteins, spike protein (S), envelope protein (E), nucleocapsid protein (N), and matrix protein (M), sixteen nonstructural proteins, and nine accessory proteins [15]. The main protease (M^pro^), papain-like protease (PL^pro^), RdRp, and spike glycoproteins are considered to play significant roles in the transcription and translation of the viral genome and ultimately lead to virus spread, so these are active targets for drug development against SARS-CoV-2 [16,17].

**Figure 1:**
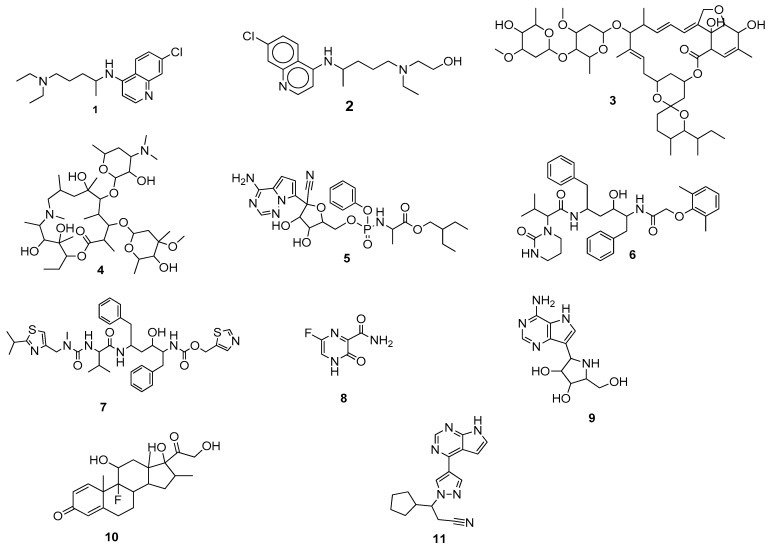
Structure of some prescribed repurposed drugs for SARS-CoV-2.

To combat the pandemic spanning 203 countries [18], different strategies were adopted, including lockdown, quarantine measures, social distancing, proper hygiene practice, face covering, and controlling sanitary conditions strictly [19]. All of these measures contributed to a reduction in normal social contacts [20]. Moreover, some already available antimalarial, immunomodulatory, and antiviral drugs (Figure 1)—chloroquine **1**, hydroxychloroquine **2**, ivermectin **3**, azithromycin **4**, remdesivir **5**, lopinavir **6**, ritonavir **7**, favipiravir **8**, galidesivir **9**, dexamethasone **10**, and ruxolitinib **11**—have been repurposed for COVID-19 treatment [21,22,23,24]. Among the above-mentioned drugs, remdesivir, lopinavir/ritonavir, and chloroquine (or hydroxychloroquine) have received increased scientific attention, but only remdesivir has been approved by the Food and Drug Administration (FDA) for the treatment of patients with COVID-19 [25].

As the number of cases grew, understanding the immune response to SARS-CoV-2 became critical. Multiple vaccines were developed, including BNT162 vaccine by Pfizer and BioNTech; mRNA-1273 vaccine by Moderna; AZD1222 by AstraZeneca and the University of Oxford; CoronaVac by Sinovac; the COVID-19 vaccine by Sinopharm and the Wuhan Institute of Virology, China; Sputnik V by the Gamaleya Research Institute, Russia; BBIBP-CorV by Sinopharm and the Beijing Institute of Biological Products, China; and EpiVacCorona by the Federal Budgetary Research Institution State Research Center of Virology and Biotechnology, Russia [26]. In the beginning, vaccine hesitancy was constituted as a threat to tackling the COVID-19 pandemic because herd immunity depended on both the availability of vaccines and the population’s willingness to accept those vaccines [27]. But later on, the efficacies of the vaccines were shown with Pfizer at 95%, Moderna at 94.1%, and AstraZeneca at 70.4%, proving that these vaccines are effective at reducing the incidence and severity of SARS-CoV-2 infection among the study populations [28].

There was an emergence of SARS-CoV-2 variants due to the changes in the nucleotides that occur naturally during replication. The SARS-CoV-2 variants of concern that have emerged till today are the Alpha variant (B.1.1.7) that was detected in the UK in September 2020, the Beta variant (B.1.351) detected in South Africa in October 2020, the Gamma variant (P.1) detected in Brazil in November 2020, the Delta variant (B.1.617.2) detected in India in December 2020, and the Omicron variant (B.1.1.529) detected in South Africa in November 2021 [29]. These circulating SARS-CoV-2 variants are challenging therapeutic actions against COVID-19.

Natural products have effectively acted as lead compounds for various infectious diseases. On this ground, various research groups across the globe working in the field of medicinal chemistry have focused on known natural products for their ability to inhibit COVID-19. We reviewed the available literature on natural compounds that show some evidence of SARS-CoV-2 inhibitory potential: first, those for which in vivo and in vitro studies were performed, next those for which a combination of in vitro and in silico studies was carried out, and finally, a wide range of compounds for which only in silico studies were conducted. The latter is divided into sections for various SARS-CoV-2 proteins and the compounds that show evidence of interaction with them.

## 2. In Vivo Studies

**Figure 2:**
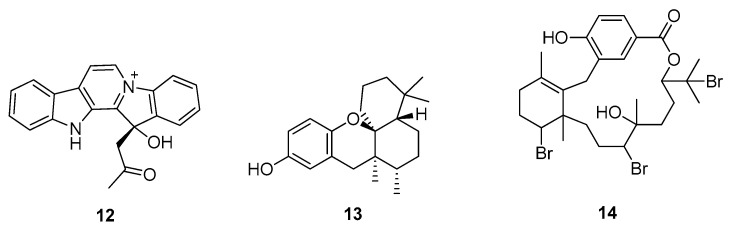
Natural compounds reported as SARS-CoV-2 inhibitors through in vivo studies.

Some marine natural products, including homofascaplysin A **12**, (+)-aureol **13**, and bromophycolide A **14** (Figure 2), have been reported as inhibitors of SARS-CoV-2 in human airway epithelial cells. SARS-CoV-2 inhibition was assessed by finding EC_50_ and CC_50_ values in human Calu-3 cells, i.e., (1.1 ± 0.4 μM, 4.0 ± 1.0 μM, and 6.9 ± 2.0 μM) and (~5 μM, >10 μM, and >10 μM), respectively, compared to those of remdesivir with EC_50_ (0.3 ± 0.0 μM) and CC_50_ (>5 μM) values [30].

## 3. In Vitro Studies

Baicalin **15** and baicalein **16** (isolated from *S. baicalensis*) were identified as inhibitors of 3-chymotrypsin-like protease (3CL^pro^), the main protease of SARS-CoV-2, through in vitro studies. The IC_50_ values were determined via a fluorescence resonance energy transfer (FRET)-based protease assay for baicalin **15** and baicalein **16**, i.e., 6.41 μM and 0.94 μM, respectively. Half-maximal effective concentrations (EC_50_) of both compounds were found to be 10.27 μM and 1.69 μM, respectively [31]. Liu et al. (2021) also tested the ethanolic extract of *S. baicalensis* and baicalein **16** for their inhibitory action on 3CL^pro^ of SARS-CoV-2. Both possessed inhibitory potentials with IC_50_ values of 8.52 μg/mL and 0.39 μM, respectively [32]. Zandi et al. (2021) reported their inhibitory potential against RdRp, the enzyme responsible for the replication of SARS-CoV-2, in Vero cells and in Calu-3 cells as well. Baicalein **16** was reported as a more potent compound with EC_50_ values of 4.5 ± 0.2 μM in Vero cells and 1.2 ± 0.03 μM in Calu-3 cells [33].

**Figure 3:**
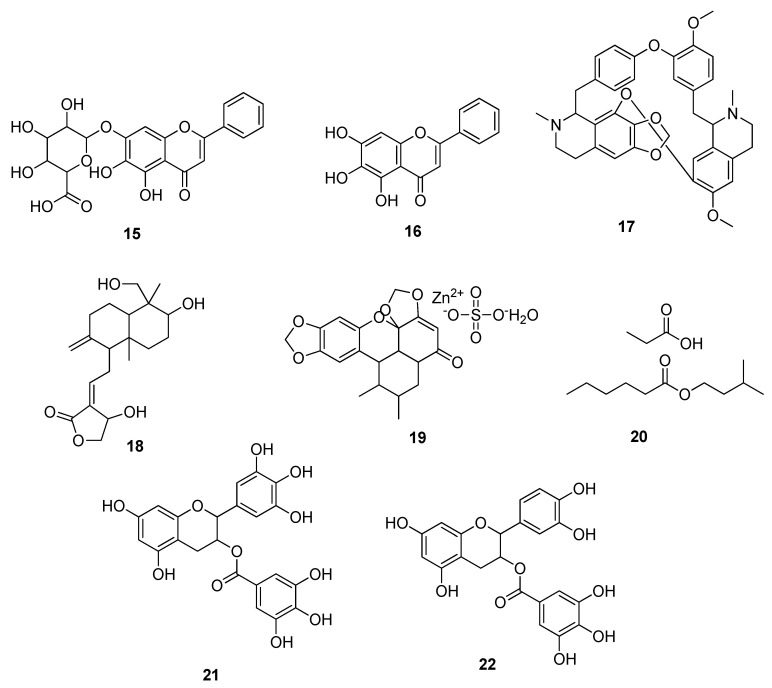
Natural compounds reported as SARS-CoV-2 inhibitors through in vitro studies.

He et al. (2021) identified cepharanthine **17**, a *bis*-benzylisoquinoline alkaloid, as the active drug candidate against SARS-CoV-2 with an EC_50_ value of 3.35 μM. Studies revealed that mechanistically, Compound **17** performed the pivotal role as a Ca-channel blocker and, hence, caused suppression in SARS-CoV-2 entry [34]. Sa-ngiamsuntorn et al. (2020) isolated andrographolide **18** from *Andrographis paniculata* and tested it in human lung epithelial cells (Calu-3) via an in vitro antiviral assay against SARS-CoV-2. The IC_50_, as determined by a plaque assay, was found to be 0.034 μM [35]. Saadh et al. (2021) demonstrated that zinc sulphate in combination with sauchinone, i.e., sauchinone/Zn-II **19**, showed more additive and inhibitory effects against 3CL^pro^ of SARS-CoV-2. Zinc, when combined with sauchinone, a well-known antiviral drug, displayed a 2.02-fold greater decrease in its inhibitory effect, i.e., the IC_50_ value of sauchinone was 4.325 μM [36]. Brown et al. (2021) identified the propylamylatin^TM^ formula as an effective inhibitor of SARS-CoV-2 by performing plaque assays in Vero E6 cells. A 90% effective inactivation potential (EC_90_) of propylamylatin^TM^ **20** was observed to be 4.28 μls, which was found to be better than the individual components (propionic acid; EC_90_ = 11.50 μls, isoamyl hexanoates; EC_90_ = 10-fold reduction in viral infection) of the mixture [37]. Liu et al. (2021) identified that the infection caused by SARS-CoV-2 was significantly suppressed by green tea beverages and their active catechin components. Among various tea catechins, epigallocatechin gallate (EGCG) **21** (Figure 3) effectively blocked viral entry into the host cell by interacting with the viral spike proteins and host ACE2 receptor (EC_50_ value = 43.48–107.6 ng/mL) with noncytotoxic doses [38].

Similarly, Tun et al. (2022) reported the inhibition of SARS-CoV-2 main protease by six types of Japanese green tea beverages and tea ingredients. These six types of tea beverages inhibited SARS-CoV-2 infection by 70–88% in a dilution-dependent way. The tea ingredients epigallocatechin gallate (EGCG) **21** and epicatechin gallate **22** (Figure 3) suppressed the antiviral activity with IC_50_ values of 12.5 and 6.5 μM, respectively. Moreover, these active compounds also interact with the viral entry into the host [39]. The antiviral potential of *Spatholobus suberectus Dunn* extract against SARS-CoV-2 infection was assessed using an in vitro analysis. The results have shown that the plant extract effectively blocks the viral spike proteins and host ACE2 cells interaction, and EC_50_ values were obtained in the range of 3.6 to 5.1 μg/mL. Further, in vivo studies showed that it could act as a potential inhibitor with no toxicity in long-term treatment [40]. Eggers et al. (2022) analyzed the inhibitory effect of various plant juices (black chokeberry, elderberry, and pomegranate) and green tea against SARS-CoV-2. Cell-based assays demonstrated a reduction in viral infection by ≥80% or ≥99%, while black chokeberry juice was found to be the most effective suppressor of SARS-CoV-2 with a percentage inhibition of 96% [41]. Baeshen et al. (2022) explored the anti-SARS-CoV-2 activity of natural extracts of the desert medicinal plant *Rhazya stricta* in a dose-dependent way. The highest activity was observed for the nonalkaloidal fraction (IC_50_: 0.0461 mg/mL; CC_50_: 0.18 mg/mL), and then weak base alkaloids had activity with an IC_50_ of 0.0474 mg/mL and a CC_50_ of 0.0464 mg/mL [42]. A natural nutraceutical, BEN815, contained extracts of green tea leaves (*Camellia sinensis*), guava leaves (*Psidium guajava*), and rose petals (*Rosa hybrida*) that displayed an antiviral effect against SARS-CoV-2 (IC_50_ value = 34.38 μg/mL). This study also showed that among the various components of BEN815, EGCG **21** demonstrated the highest inhibitory potential with an IC_50_ of 33.41 μM [43].

## 4. In Vitro and In Silico Studies

Khan et al. (2021) confirmed that kaempferol **23** exhibited good inhibitory activity against 3CL^pro^ of SARS-CoV-2 in vitro as well as through docking analysis. The inhibitory role played by kaempferol **23** on SARS-CoV-2 was found to be 34.46 μM in Vero E6 cells [44]. Alhadrami et al. (2021) demonstrated cnicin **24** as the potential inhibitor of the nonstructural proteins, RdRp (NSP12), ADPRP (NSP3), and endoribonuclease (NSP15) of SARS-CoV-2. The inhibition potency was validated by an IC_50_ value of 1.18 μg/mL and a CC_50_ value of 59.66 μg/mL. The selectivity index (CC_50_/IC_50_) was found to be 70.3. In vitro testing confirmed the results of the molecular docking simulations [45]. Li et al. (2021) evaluated some aromatic sesquiterpenoids as SARS-CoV-2 spike–ACE2 interaction inhibitors through an in vitro analysis, and their binding interactions were studied through molecular docking studies. Among the studied compounds, candinone sesquiterpenoids **25** showed the lowest IC_50_ value, i.e., 64.5 ± 1.8 μM. Their structure–activity relationship (SAR) has shown that the pharmacophore of candinone sesquiterpenoids **25** might be the hydroxyl and carboxyl groups [46]. Hijikata et al. (2021) evaluated cepharanthine **17** through in vitro as well as in silico studies. In vitro analysis revealed that the inhibitory concentration (IC_50_) of cepharanthine **17** against SARS-CoV-2 by interacting with the human lysosomal membrane protein Niemann–Pick type C intracellular cholesterol transporter 1 (NPC1) was found to be 1.90 μM. The diphenyl ester moiety was suggested as the pharmacophore of cepharanthine **17**. Molecular docking was also carried out on AutoDock 4 (AD4) and Autodock Vina (ADV) software, and the root mean square deviation (RMSD) value obtained between the best docking poses was 1.3 in the S1 pocket of NPC1 [47]. Dogan et al. (2021) isolated artemisinin **26** from *Artemisia annua* L. and evaluated it against the M^pro^ and spike proteins of SARS-CoV-2 through in vitro and in silico evaluations. Artemisinin **26** showed maximum inhibitory potency, i.e., >200 μM, against SARS-CoV-2 in the HEK293T cell line [48]. Owis et al. (2021) isolated a flavonoid, mauritianin **27**, from *Salvadorapersica* L. and checked its inhibition (IC_50_ and CC_50_) towards 3CL^pro^ of SARS-CoV-2, which were found to be 8.59 ± 0.3 μg/mL and 24.5 ± 1.9 μg/mL, respectively [49]. Morita et al. (2021) reported that all-trans retinoic acid **28** exhibited anti-SARS-CoV-2 activity against 3CL^pro^ through Alpha screening and in vitro approaches. AlphaScreen software provided high-throughput screening of the active compounds. Further, a FRET assay was carried out, and it was noticed that the IC_50_ value obtained was 24.7 ± 1.65 μM. It **(28)** inhibited the replication of Vero E6 cells and Calu-3 cells with IC_50_ values of 2.69 ± 0.09 μM and 0.82 ± 0.01 μM, respectively [50].

**Figure 4:**
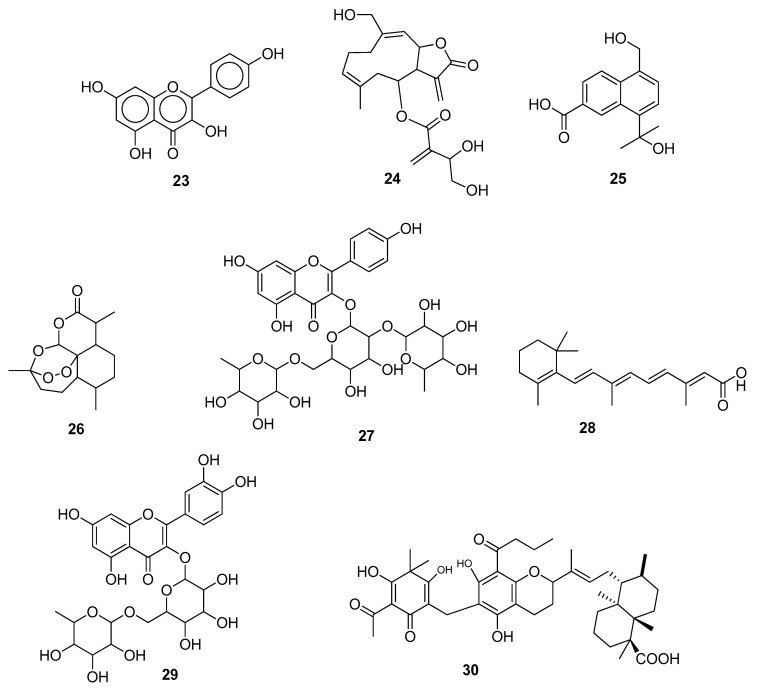
Natural compounds reported as SARS-CoV-2 inhibitors through in vitro and in silico studies.

**Figure 5:**
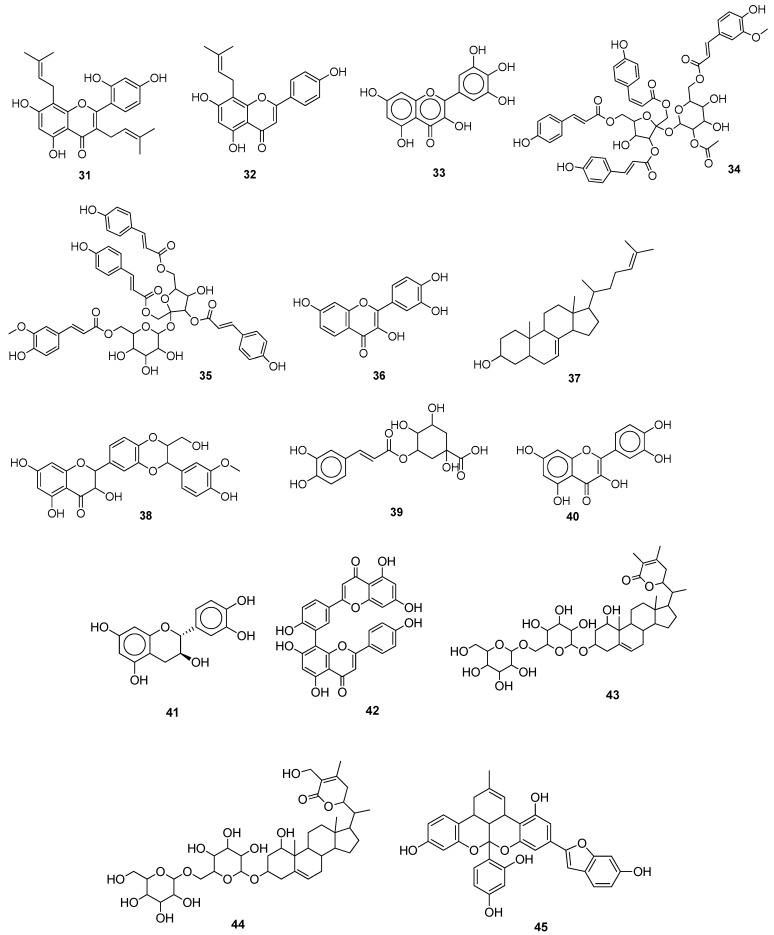
Natural compounds as anti-SARS-CoV-2 agents.

Gizawy et al. (2021) reported some bioactive compounds extracted from *Pimentadioica* (L.) as anti-SARS-CoV-2 M^pro^ agents. Rutin **29** appeared to be the best active compound, as evident from both in silico and in vitro studies. The antiviral and cytotoxic activities were determined by IC_50_ and CC_50_ values, i.e., 31 μg/mL and 8017 μg/mL, respectively [51]. In silico observations of Compounds **23**–**28** and **30** (Figure 4) are depicted in (Table 1). Hou et al. (2022) identified phloroglucinols terpenoids as the target inhibitors of 3CL^pro^ of SARS-CoV-2 through virtual screening and in vitro approaches. The terpenoid **30**, isolated from *Dryopteris wallichiana*, showed the best inhibitory activity in Vero E6 cells with an effective inhibitory concentration of 4.5 μM. Compound **30** was virtually screened on AutoDock 4 to see the binding interactions between the ligand and amino acid residues of 3CL^pro^. It was noted that Compound **30** formed H-bonds with MET165, ASN142, GLU166, and GLN192 and hydrophobic interactions with PHE140 [52]. Kim et al. (2022) identified the antiviral potential of mulberrin (kuwanon C) **31** (Figure 5), isolated from the commonly known mulberry plant. In vitro studies showed that this natural compound inhibited the SARS-CoV-2 infection by interacting with both the viral spike proteins and the host ACE2 receptor cells with an IC_50_ value of 91.4 μM at a concentration range of 25 μM to 100 μM. Furthermore, in silico studies demonstrated the more stable binding interactions of ligands with spike proteins compared to ACE2 receptors [53]. Some naturally occurring flavonoids (myricetin, quercetin, kaempferol, flavanone, and licoflavone C) were found to inhibit the enzymatic activities of SARS-CoV-2 by selectively antagonizing the action of NSP13 at nanomolar and micromolar concentrations. However, computational analysis has shown that among these active compounds, licoflavone C **32** (Figure 5) displayed the highest affinity and strong binding interactions with amino acid residues, such as LYS569, GLY538, HIS290, and ARG442, followed by myricetin, baicalein, kaempferol, and quercetin [54].

Xiao et al. (2021) investigated the inhibitory activity of 15 natural compounds (flavonoids, coumarins, terpenoids, phenolics, aldehydes, and steroids) towards the SARS-CoV-2 main protease using enzymatic and virtual screening analysis. Among them, myricetin **33** had the most potent activity with an IC_50_ of 3.684 ± 0.076 μM. Molecular docking showed interactions with the M^pro^ binding pocket via the chromone ring and its 3′-, 4′-, and 7-hydroxyl groups [55]. Compounds **34** and **35** and extracts of *R. japonica* and *Reynoutriasachalinensis* species were evaluated against the main protease of SARS-CoV-2 through in silico and in vitro studies. The compounds vanicoside A **34** and vanicoside B **35** showed the best results towards inhibition of the targeted enzyme (M^pro^), having an IC_50_ of 23.10 μM and 43.59 μM, respectively. Plant butanol fractions demonstrated the greatest suppression of SARS-CoV-2’s M^pro^ (the IC_50_ values for *R. sachalinensis* and *R. japonica* were 4.031 g/mL and 7.877 g/mL, respectively [56]. Chaves et al. (2022) analyzed the anti-SARS-CoV-2 potential of some classes of flavonoids: flavonol (fisetin, kaempferol, myricetin, and quercetin), flavone (apigenin and luteolin), and isoflavone (genistein). It was found that flavonols had better inhibitory potential than flavones and isoflavones, and this was observed due to the presence of a greater number of hydroxyl groups in the B ring of flavonols. Among flavonols, fisetin **36** and myricetin **33** mainly targeted the main protease with EC_50_ values of 2.03 ± 0.10 μM and 0.91 ± 0.05 μM, respectively. Furthermore, in silico analysis confirmed the better inhibitory potential of these flavonols compared to other flavonoids [57]. Hafez et al. (2022) isolated the natural compound **37** from *Ophiocoma dentata* (brittle star) and proposed it as an active inhibitor of SARS-CoV-2’s M^pro^, NSP10, and RdRp through in vitro and in silico studies. An effective dose of 12.48 μM demonstrated 95% inhibition, and Compound **37** displayed an IC_50_ of 11,350 ± 1500 ng/mL against normal fibroblast cells. Moreover, in silico analysis provided strong binding interactions between the studied compounds and targeted viral enzymes [58]. Silibinin **38**, a naturally occurring flavonoid, suppressed SARS-CoV-2 infection by inhibiting the main protease, S-proteins, and RdRp with IC_50_ values of 0.021, 0.029, and 0.042 μM, respectively. It showed >90% inhibition of viral activity at a concentration of 0.031 μM. Moreover, in silico studies showed that this potent compound showed good binding affinity with both spike proteins and the main protease [59]. Elhusseiny et al. (2022) analyzed the antiviral potential of aqueous extracts of *Agaricus (A.) bisporus*, *Lentinula (L.) edodes*, and *Pleurotus (P.) ostreatus* against SARS-CoV-2’s main protease, with IC_50_ values of 10.3.3, 26.17, and 39.19 μg/mL, respectively. Furthermore, docking analysis showed that chlorogenic acid **39**, kaempferol **23**, quercetin **40**, and catechin **41** were the most active compounds that were bound effectively with binding interactions ranging between −22.8 and −37.61 kcal/mol [60]. Lopes et al. (2022) isolated 55 bioactive plant compounds and evaluated their potential against the M^pro^, RdRp, PL^pro^, NSP15, endoribonuclease, spike protein, and ACE2 using molecular docking and in vitro analysis. The docking results demonstrated that 7-*O*-galloylquercetin, amentoflavone, gallagic acid, and kaempferitrin displayed good binding interactions with the targeted enzymes of SARS-CoV-2. Among these, amentoflavone **42** suppressed the activity of 3CL^pro^ with an IC_50_ value of 8.3 μM [61]. Nine withanolides from *Ashwagandha* were found to cause inhibition of transmembrane serine protease 2 (TMPRSS2) (a human gene) and the M^pro^ of SARS-CoV-2. Among these nine tested compounds, the best binding affinities were observed for withanoside V **43** and withanoside IV **44,** which formed hydrogen bonds and other interactions with targeted proteins. Additionally, in vitro analysis through a 3-(4,5-dimethylthiazol-2-yl)-2,5-diphenyl-2*H*-tetrazolium bromide (MTT) assay confirmed the antiviral potential of selected compounds at a concentration >10 mM [62]. Kim et al. (2022) identified the antiviral potential of mulberrofuran G **45** (Figure 5) isolated from the commonly known mulberry plant. In vitro studies showed that this natural compound inhibited the SARS-CoV-2 infection by interacting with both the viral spike proteins and the host ACE2 receptor cells with an IC_50_ of 10.23 μM at a concentration of less than 50 μM. Furthermore, in silico studies demonstrated the more stable binding interactions of ligands with spike proteins compared to ACE2 receptors [63] (Table 1).

Remdesivir was observed to be the most frequently used control group in many in vitro studies reported in the literature. Significant IC_50_ and EC_50_ values observed for remdesivir fell in the range of 0.086–13.1 μM [31,33,34] and 0.77–5 μM [35,43,47,59], respectively. Naturally occurring Compounds **16** (EC_50_ = 1.69 μM [31]), **17** (IC_50_ = 1.90 μM [47] and EC_50_ = 3.35 μM [34]), **18** (IC_50_ = 0.034 μM [35]), and **38** (IC_50_ = 0.021 μM [59]) showed the most potent inhibitory activity. Moreover, Compounds **15** (EC_50_ = 10.27 μM [31]) and **21** (IC_50_ = 33.41 μM [43]) showed moderate potential to inhibit SARS-CoV-2.

**Table 1:** In silico results for compounds for which in vitro studies were also performed.

Serial No.	Compound	Binding Potential		Interactions at Enzyme Active Site *		References
		Docking Score (kcal/mol)	Binding Free Energy (kcal/mol)	H-Bond Interactions	Hydrophobic Interactions	
**Main Protease (M^pro^/3CL^pro^)**						
**1**	Kaempferol **(23)**	−6.4	−26.81	PHE140, LEU141, ASN142, **CYS145**, ARG188	-	[44]
**2**	Artemisinin **(26)**	−5.21	−25.84	ASN142	LEU141, **CYS145**, VAL42, CYS44, MET49, LEU27, MET106	[48]
**3**	Rutin **(29)**	−9.19	-	HIS163, GLU166(2), PHE140, **CYS145**	**HIS41**	[51]
**4**	Myricetin **(33)**	<−8.0	-	PHE140, GLU166, ASP187	**HIS41**	[55]
**5**	5α-cholesta-4 (27), 24-dien-3β, 23 β-diol **(37)**	-	−24.68	**HIS41**	MET49, MET165	[58]
**6**	Chlorogenic acid **(39)**	-	−24.9	GLY143, **CYS145**	MET165	[60]
**7**	Kaempferol **(23)**	-	−36.5	GLU166, MET49	**HIS41**, **CYS145**	[60]
**8**	Quercetin **(40)**	-	−39.66	GLU166, GLY143, **CYS145**	MET49	[60]
**9**	Catechin **(41)**	-	−39.66	GLU166, ASN142, HIS163, PHE140	MET165, LEU141	[60]
**10**	Amentoflavone **(42)**	−8.7	-	-	**CYS145**	[61]
**Papain-Like Protease (PL^pro^)**						
**11**	Amentoflavone **(42)**	−7.7	-	-	TYR264	[61]
**Spike Proteins**						
**12**	Candinone sesquiterpenoids **(25)**	-	-	ARG403, ARG405, ARG408, ARG393, HIS34	GLU406, LYS417, ARG403, GLN409	[46]
**13**	Artemisinin **(26)**	−5.06	−30.61	GLU23, LYS26, THR27, ASP30, LYS417, TYR421, PHE456, ARG457, TYR473		[48]
**14**	Mauritianin **(27)**	-	−9.4799	ASP405, GLY496, LYS403, GLU37, ASP30	HIS34, ALA387	[49]
**15**	Kuwanon C **(31)**	−7.1	-	ARG403	TYR453, GLN493, SER494, TYR495, GLY496, PHE497, GLN498, ASN501, GLY502, TYR505	[53]
**16**	Silibinin **(38)**	−7.78	-	ASN907, LYS1038, ILE909, GLU1092, TYR904, ASN907, GLY904, TYR904	TYR904, ASN907, GLY910, GLY908, LYS1038, GLU1092, TYR904	[59]
**17**	Amentoflavone **(42)**	-8.7	-	-	TYR449, GLN493	[61]
**18**	Mulberrofuran G **(45)**	−8.4	-	GLN493, TYR453 GLY496	GLU406, LYS417, LEU455, SER494, TYR495, GLY496, GLN498, ASN501, TYR505	[63]
**(a) ACE2**						
**19**	Amentoflavone **(42)**	−9.1	-	-	ASP30, ASP32, GLN42, TYR83, LYS353	[61]
**20**	Mulberrofuran G **(45)**	−7.4	-	GLU23, THR27, ASN33, GLN96	GLU23, GLN24, LYS26, THR27, LEU29, ASP30, VAL93, AND PRO389	[63]
**(b) TMPRSS2**						
**21**	Withanoside V **(43)**	−7.96	-	GLU299, TYR337, SER339	HIS296, LYS300, TYR337, ASP338, LYS340, THR341, LYS342, ASP435, SER436, CYS437, GLN438, GLY439, SER441, THR459, SER460, TRP461, GLY462, GLY464, CYS465, GLY472, VAL473	[62]
**22**	Withanoside IV **(44)**	−6.92	-	ASP338, LYS342, GLU389, SER436, SER441	HIS296, GLU299, TYR337, ASP435, CYS437, GLN438, GLY439, ASP440, THR459, SER460, TRP461, GLY462, GLY464, CYS465, ALA466, GLY472, VAL473	[62]
**RDRP**						
**23**	Cnicin **(24)**	−9.7	−10.3	LYS41, LEU49, LYS50, THR51, ASN52	PHE35, ASP36, ILE37, TYR38, ASN39, VAL42, PHE48, HIS75, ASP208, ASP218, ASP221	[45]
**24**	5α-cholesta-4(27), 24-dien-3β, 23 β-diol **(37)**	-	−29.86	TR680, CYS622	URD20, ADE11, LYS545, URD10, VAL557, ALA547	[58]
**25**	Silibinin **(38)**	−7.15	-	U20, ASP618, ILE548, ASP618	ARG836, ASP618, SER814, GLU811, LYS545, LYS551, ALA547, **ASP760**, ILE-548	[59]
**26**	Amentoflavone **(42)**	−9.4	-	-	ASP618	[61]
**Endoribonuclease**						
**27**	Cnicin **(24)**	−9.8	−9.3	GLN245, **HIS250**, LYS290	ASN278, VAL292, SER294, **THR341**, TYR343, PRO344, LYS345, LEU346	[45]
**Adeno Diphosphate (ADP)-Ribose Phosphatase/ADPRP**						
**28**	Cnicin **(24)**	−9.2	−10.1	ALA38, VAL49, ALA50, LEU126, ALA129, ILE131, PHE132, ALA154, PHE156, ASP157	ALA21, ILE23, GLY47, PRO125, SER128, LEU160	[45]
**Nonstructural proteins**						
**29**	5α-cholesta-4(27),24-dien-3β,23-β-diol **(37)**	-	−23.47	ASP6912	LEU6898, PRO6932	[58]

* Catalytic site residues are shown in blue, and binding site residues are shown in black in Table 1.

## 5. In Silico Studies

### 5.1. Natural Compounds as SARS-CoV-2 Main Protease Inhibitors

Recent studies have shown that during their life cycle, coronaviruses typically accumulate a few polypeptides and then develop proteolytic breakdown to produce an additional 20 proteins [11]. Among these proteins, it is highlighted that the main protease (M^pro^/3CL^pro^) and papain-like protease (PL^pro^) play a crucial role in the transcription and replication of viruses. These proteases have been the subject of significant research to find specific COVID-19 inhibitors. Computational studies using various in silico approaches made it possible to find potential inhibitors against viral proteases.

**Figure 6:**
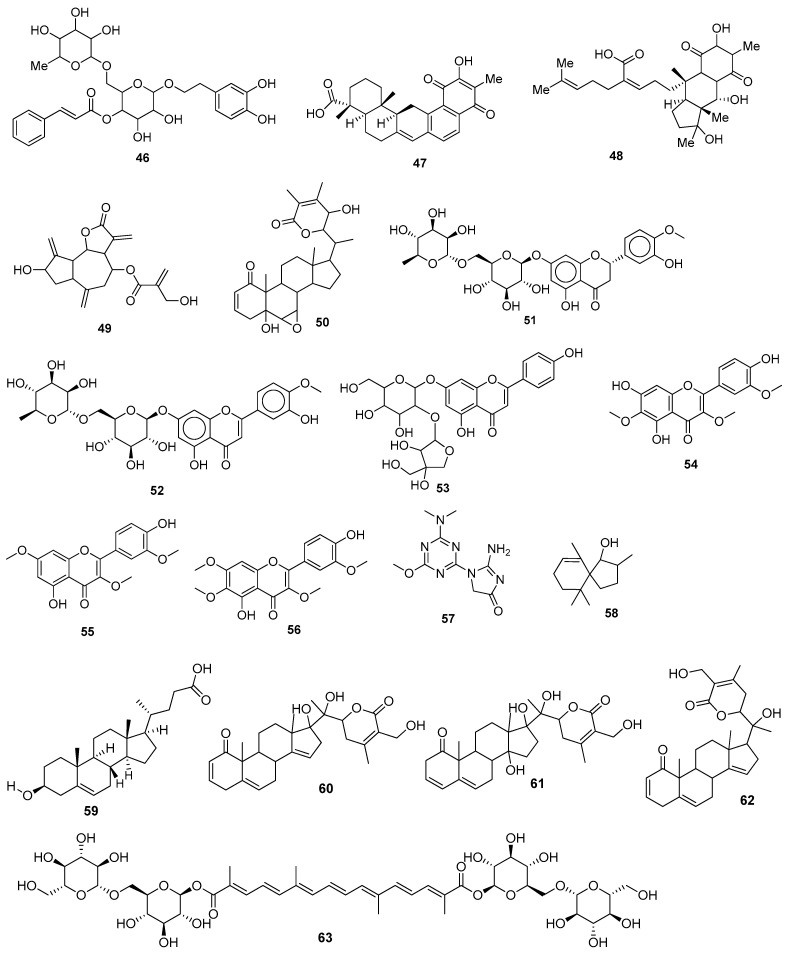
Natural compounds as M^pro^ inhibitors.

Bernardi et al. (2021) used a computational virtual screening approach to investigate phenylethanoid glycosides as inhibitors of SARS-CoV-2’s main protease (M^pro^). Three major phenylethanoid glycosides, forsythiaside A **46** (Figure 6), isoacteoside, and verbacoside, were studied due to their good docking scores and strong binding interactions at the active site [64]. (The docking scores and amino acid residues are listed in Table 2).

**Figure 7:**
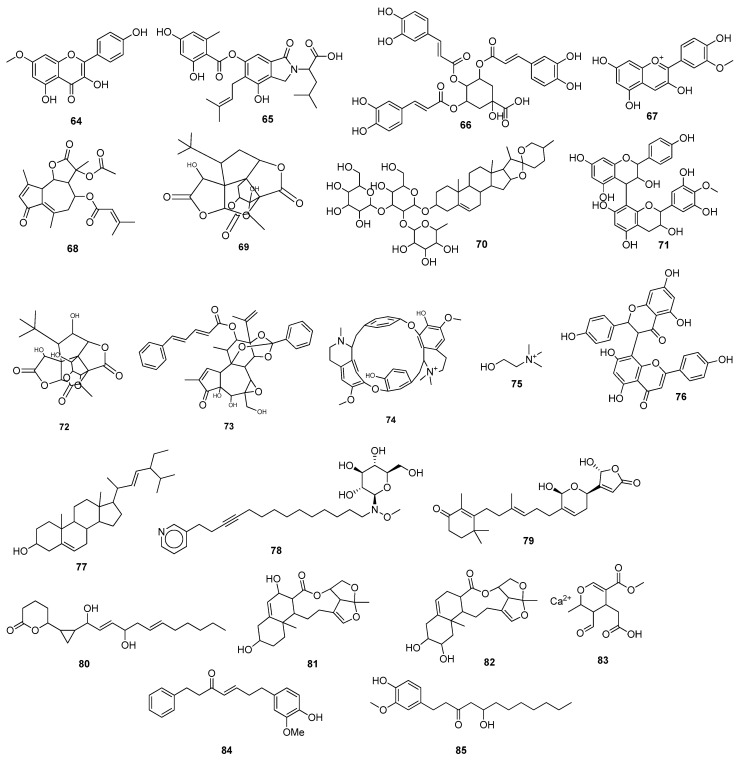
Natural compounds acting as anti-COVID-19 agents by prohibiting M^pro^.

Some potential phytochemicals from Cameroonian plants and bioactive lactones from *Saussureacostus* were investigated to combat SARS-CoV-2’s M^pro^. ADMET (absorption, distribution, metabolism, excretion, and toxicity) analysis was also carried out to check the pharmacological properties of pycnanthuquinone C **47** (Figure 6) and pycnanthuquinone A **48**, which were extracted from *Pycnanthusangolensis* [65]. According to the visualization results, the interaction between the M^pro^ and cyanropicrin **49** was the best one, as proved by the molecular docking (MD) simulation study. The drug-likeness of cyanropicrin **49** was confirmed using ADMET analysis and Lipinski’s rule [66]. The phytocompounds from Indian medicinal plants and some polyphenols were examined as potential inhibitors of SARS-CoV-2’s main protease. The studies revealed that withanolide R **50** had the lowest relative free binding energy value and was declared to be the most potent among the studied compounds [67]. Eighty polyphenols were initially tested, and four of them—hesperidin **51**, rutin **29**, diosmin **52**, and apiin **53**—showed active inhibitory activity against the M^pro^ [68]. Phytochemicals from Jordanian hawksbeard, jaceidin **54**, pachypodol **55**, and chrysosplenetin **56** showed good binding affinities to the main protease (M^pro^) of SARS-CoV-2 [70]. Imidazoline-4-one-2-imino-1-(4-methoxy-6-dimethylamino-1,3,5-triazin-2-yl) **57**, spiro[4,5]dec-6-en-1-ol, 2,6,10,10-tetramethyl **58**, and 3-hydroxy-5-cholen-24-oic acid **59** extracted from *Tinosporacrispa* showed the best binding affinities against the M^pro^ [71]. Withacoagulin H **60,** ajugin E **61**, withacoagulin **62** [72], crocin **63** (Figure 6) [73], rhamnocitrin **64** (Figure 7) from *Artemisia annua* [74], and sterenin M **65**, a fungal metabolite [75], were shown to be the best active compounds against the M^pro^. Rhamnocitrin **64** also possessed a favorable ADMET profile with no hepatotoxicity, carcinogenicity, mutagenicity, cytotoxicity, and immunotoxicity [74]. The inhibitory activity of honeybee natural products and some natural flavonoids and peonidin was studied against SARS-CoV-2’s main protease (M^pro^) through an in silico evaluation. It was found that 3,4,5-tricaffeoylquinic acid **66** possessed the highest binding affinity value [76]. Quercetin **40** and the peonidin **67** moiety were shown to have inhibitory potency against the M^pro^. Djiboutian medicinal plants and some natural products have potential as inhibitors of SARS-CoV-2’s M^pro^. Nine molecules have been studied, out of which rutin **29,** catechin **41,** and kaempferol **23** showed the best binding affinities with the M^pro^ compared to the reference drug, remdesivir (−7.194 kcal/mol) [69], whereas amentoflavone **42** was found to be the best active compound compared to the reference drug hydroxychloroquine with a binding free energy value of −6.3 kcal/mol. Lipinski’s rule of five also showed its drug-like properties. Amentoflavone **42** can be found in many medicinal plants, such as *Selaginellaceae*, *Cupressaceae*, and *Euphorbiaceae* family plants [78]. (The binding affinities and amino acid residue interactions are depicted in Table 2).

Out of 10 active compounds found in *Aloe vera*, ferolide **(68)** (Figure 7) was demonstrated as the most potent compound against a viral protein, i.e., 3CL^pro^, an enzyme that plays a key role in post-translational protein regulation, particularly the cleavage of viral polyproteins into functional protein units. According to the results of virtual screening, it was observed that ferolide **68** also followed Lipinski’s rule of five to be used as a drug [80]. The terpenoid ginkgolide A **69**, extracted from *Ginkgo biloba* [81], showed the highest S-score. Gracillin **70** extracted from *Paris vietnamensis* and proanthocyanidin **71** extracted from *Cinnamomum* sp. showed the lowest docking score compared to the reference drug, boceprevir (−7.7 kcal/mol) [82]. Ginkgolide M **72**, mezerein **73**, and tubocuraine **74** showed the best binding affinities against 3CL^pro^ compared to the reference drugs nelfinavir and lopinavir, with −9.1 kcal/mol and −8.4 kcal/mol binding affinities, respectively [83]. Choline **75** exhibited drug-like properties approved via the Lipinski, Veber, and Egon rules. The pharmacokinetic study revealed that it also showed gastrointestinal absorption [84]. The biflavonoids amentoflavone **42** and volkensiflavone **76** displayed the highest binding affinity to 3CL^pro^.The biflavonoid amentoflavone **42** also exhibited the highest binding affinity to PL^pro^ [79]. Jamhour et al. (2021) tested thirty-six phytochemicals under an in silico perspective; six (rutin, quercetin, catechin gallate, rhamnetin, campesterol, and stigmasterol) out of 36 were found to be bioactive. Stigmasterol **77** had the lowest binding energy value of −6.30 kcal/mol against 3CL^pro^ [85]. Hesperidin **51** showed the highest binding affinity to 3CL^pro^ compared to the standard drugs nelfinavir, hydroxychloroquine sulphate, and chloroquine [86]. Natural compounds extracted from *Amphimedon* sp. were investigated through a computational study. Amphimedoside C **78** was found to be the most active ligand against 3CL^pro^ of SARS-CoV-2 [87]. The marine-derived bioactive compound fasciospongide A **79** was found to be the most active compound against 3CL^pro^, compared to the reference drugs lopinavir and ritonavir, which had molecular mechanics Poisson–Boltzmann surface area (MM/PBSA) scores of −101.13 kJ/mol and −97.40 kJ/mol, respectively. Constanolactone B **80** was revealed to be the most bioactive compound with a lower MM/PBSA score against PL^pro^ compared to the reference drugs lopinavir and ritonavir, which had binding free energy values of −60.84 kJ/mol and −70.74 kJ/mol, respectively [88]. Glaucogenin D **81** was found to be the best active compound against 3CL^pro^, whereas glaucogenin D **81** and glaucogenin A **82** were observed to have the best binding affinity towards papain-like protease (PL^pro^) [89]. Mehmood, A. et al. (2021) identified the Quranic and prophetic medicinal plants capable of inhibiting SARS-CoV-2’s essential enzymatic functions of 3CL^pro^, the viral main proteinase, which was inhibited by calcium elenolate **83** [90]. (*E*)-7-(4-hydroxy-3-methoxyphenyl)-1-phenylhept-4-en-3-one **84**, extracted from *Alpinia officinarium*, showed the lowest binding energies and the highest ligand efficiency values in closed (−47 kJ/mol, 0.49) and open (−28 kJ/mol, 0.27) conformations, respectively. The 8-gingerol **85** (Figure 7) from ginger showed the best binding energies and ligand efficiency values in closed (−43 kJ/mol, 0.45) and open (−15 kJ/mol, 0.16) conformations, respectively, [124] (as depicted in Table 2).

**Figure 8:**
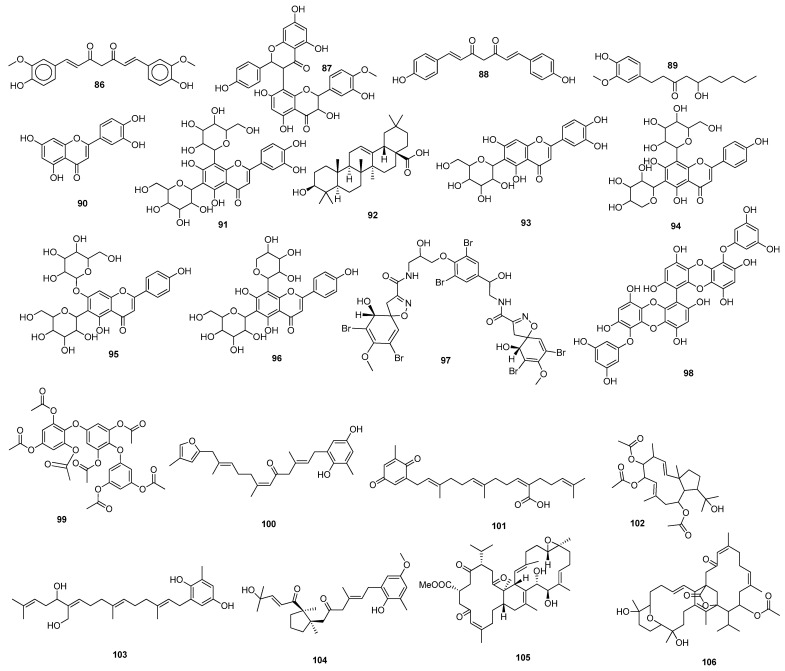
Chemical structures of SARS-CoV-2 main protease inhibitors.

Abodunrin et al. (2022) examined the therapeutic functions of the active chemicals found in ten common African medicinal herbs. Five active compounds, including curcumin **86** (Figure 8), kolaviron **87**, bisdemethoxycurcumin **88**, 6-gingerol **89**, and artemisinin **26**, were chosen and docked against the main protease. The results of the pharmacokinetic prediction showed that none of these five active substances exhibited any affinity for cytotoxicity, hepatotoxicity, mutagenicity, or carcinogenicity, maintaining their excellent ADMET profile [94]. A total of 29 compounds were isolated from the medicinal plant *Passiflora*, and all showed comparable binding affinity with various amino acid residues of the main protease. Compounds, such as luteolin **90**, lucenin **91**, olealonic acid **92**, isoorientin **93**, isochaphoside **94**, saponarin **95**, and schaftoside **96,** were bound above −8.0 kcal/mol binding energy (Figure 8). In addition to ADMET, Lipinski, Veber, and Ghose criteria were used to investigate the drug-like characteristics of *Passiflora* chemical compounds [95]. The molecular docking results showed that Compound **97** (CID 11170714) (C_31_H_30_Br_6_N_4_O_11_), which belongs to the family *Aplysinidae*, showed a good docking score and better binding interactions. MD simulation (RMSD and RMSF) studies have also been performed, which reflect a stable binding contact between the ligand and the target enzyme, M^pro^ [149]. Later on, 1018 natural brown algal compounds were taken from the COVID-19 major protease-screening database MarinLit, which is devoted to marine natural materials. The interactions between the top seven chemicals (7,2″-bieckol **98**, 7-hydroxyeckol-hepta-acetate **99**, 5-hydroxy-cystofurano-quinol **100**, sargaquinoic acid **101**, triacetoxy-18-hydroxy-2,7-dolabelladiene **102**, fallahydroquinone **103**, and methoxybifurcarenone **104** (Figure 8)) and the active site of the M^pro^ were investigated in more detail. Compound **98** displayed the lowest binding energy (high binding affinity) among all the compounds under investigation [96] (Table 2).

**Figure 9:**
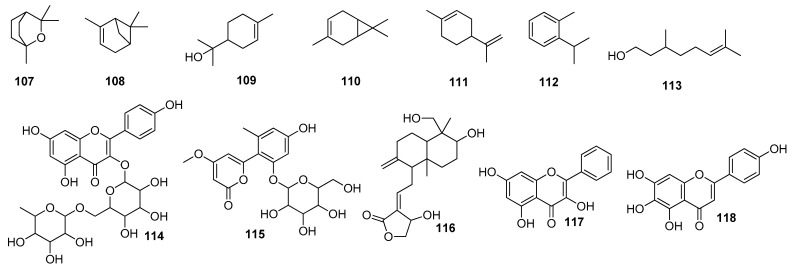

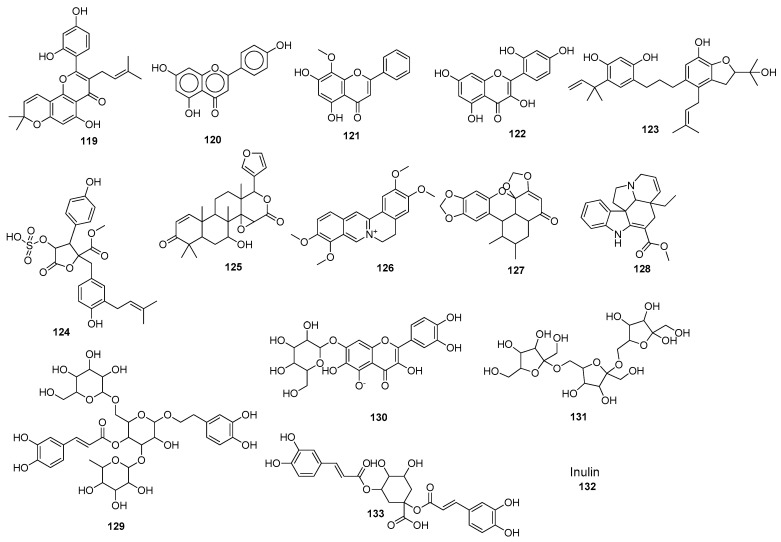
SARS-CoV-2 M^pro^ inhibition from natural sources.

Ibrahim et al. (2021) screened 360 metabolites (cembranoid diterpenes) from the genus *Sarcophyton* (soft coral) against the M^pro^ of SARS-CoV-2. Almost 59 compounds showed better docking scores compared to the docking score of the reference (darunavir = −8.2 kcal/mol). Sarelengan B **105** possessed the highest docking score, followed by bislatumlide A **106** (Figure 8). Further, from the MD simulation studies and molecular mechanics with generalized Born and surface area salvation (MM/GBSA) binding energy calculation results, it was found that Compound **105** showed favorable binding affinity with ∆G binding of <−44.0 kcal/mol against the target protein. Drug-likeness studies also demonstrated convenient physicochemical properties for Compound **106** (Figure 8) [97]. The molecular interactions of bioactive metabolites from the oils of *Eucalyptus* and *Corymbia* against SARS-CoV-2 were assessed. The compounds that showed excellent ADMET profiles and properties of drug-likeness included citronellol, α-terpineol, *o*-cymene, *d*-limonene, eucalyptol, α-pinene, and 3-carene. In addition to this, the primary component in the leaves of *Eucalyptus globulus* essential oil, eucalyptol **107** (Figure 9), had good bioavailability and blood–brain barrier (BBB) penetration and was an inhibitor of CYP 2C9 and CYP 3A4, as well as being noncarcinogenic in rat and mouse studies. Eucalyptol **107** showed less binding energy than α-pinene **108**, α-terpineol **109**, 3-carene **110**, *d*-limonene **111**, *o*-cymene **112**, and citronellol **113** (Figure 9). Based upon these results, these bioactive substances may work as potential inhibitors of virus replication and transcription by inhibiting the M^pro^ [98]. Compounds from 10 different species of medicinal plants were isolated and investigated for their therapeutic potential against the SARS-CoV-2 main proteases. The results of biological activity, pharmacological behavior, and binding affinities showed high absorption and bioavailability for *harsingar*, *aloe vera*, and *giloy* plants. Other active SARS-CoV-2 protease inhibitors included turmeric, neem, and ginger. All these plants showed more inhibition potential compared to chloroquine and hydroxychloroquine. The docking results demonstrated the highest inhibition potential for the extracts of *harsingar* and *aloe vera*, namely, nictoflorin **114** and aloenin **115** (Figure 9) [99]. Vijayakumar et al. (2022) investigated the therapeutic potential of phytochemicals present in a medicinal herb named *Andrographis panniculata* (*A. panniculata*). Five diterpenoid molecules, andrographolide, neoandrographolide, 14-deoxyandrographolide, 14-deoxy-11, 12-didehydroandrographolide, and andrograpanin, extracted from *A. panniculata* were screened. The results from pharmacokinetics and molecular dynamic simulation showed that all the selected diterpenoids possessed a significant inhibitory potential against the M^pro^ of SARS-CoV-2. Among these bioactive compounds, andrographolide **116** showed a high potential for M^pro^ inhibition [100]. (Binding affinities, binding free energies, and amino acid residue interactions are depicted in Table 2).

Flavonoids are naturally occurring phytochemical compounds that possess various biological applications, including antiviral activities. Bora et al. (2021) assessed the inhibitory potential of four naturally occurring flavonoids (quercetin, luteolin, galangin **117**, and naringenin) against the SARS-CoV-2 main protease. All four compounds—quercetin, luteolin, galangin, and naringenin—exhibited satisfactory docking scores. Among these four, galangin **117** displayed the highest number of interactions with a large number of amino acids. Furthermore, the pharmacokinetic results revealed that galangin could be more potent against SARS-CoV-2’s M^pro^ [101]. Moreover, the flavonoids, including amentoflavone **42**, scutellarin **118**, morusin **119**, apigenin **120**, wogonin **121**, kaempferol **23**, fisetin **36**, kuwanon C **31**, and morin **122**, showed strong binding interactions with the M^pro^ at several amino acid residues. In addition to these, prenylated flavonoids, flavanones, bioflavonoids, flavones, and flavan-3-ols demonstrated good interactions and binding affinities with the COVID-19 main protease [102]. Among the 2360 natural compounds, 12 compounds from different natural sources (microbes, fungi, and plants) showed better docking results below −12 kcal/mol. Kazinol T **123** (discovered from *Broussonetiakazinoki*) exhibited the highest score, followed by butyrolactone 1,3 sulphate **124** (Figure 9) (extracted from *Aspergillus terreus*). ADMET analysis predicted no considerable toxicity for the active lead compounds [103] (Table 2).

**Figure 10:**
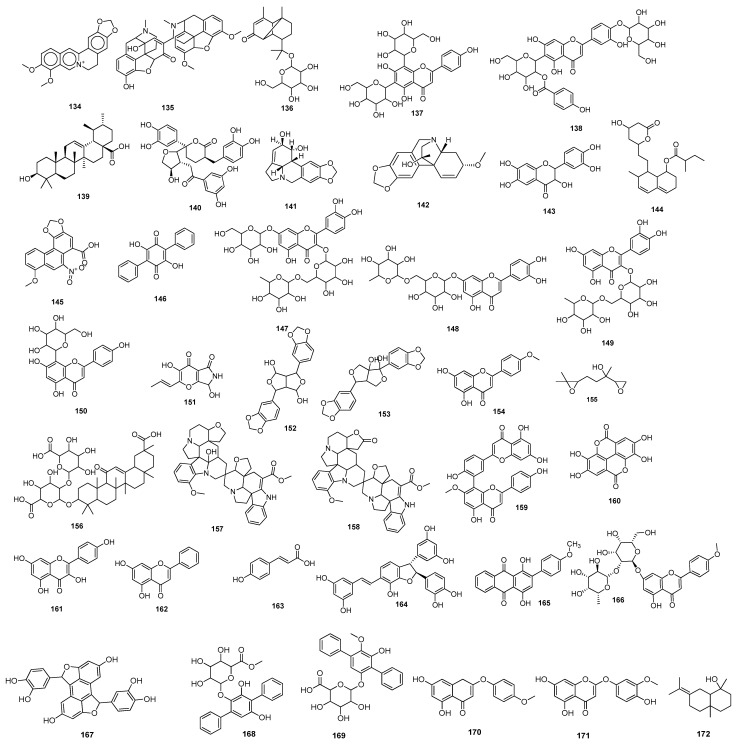
Chemical structures of potent inhibitors of M^pro^.

Baildya et al. (2021) predicted the inhibitory potential of neem extracts containing 19 natural compounds on the PL^pro^ of SARS-CoV-2. All the extracted compounds showed satisfactory inhibitory potential against the target enzyme. Among them, desacetyl gedunin **125**, which was extracted from neem seed, exhibited the highest binding affinity towards PL^pro^. However, the ADMET analysis and pharmacokinetic studies confirmed high blood–brain barrier permeability, bioavailability, and low toxicity of selected compounds compared to the standard drugs [104]. The binding affinities of palmatine **126**, sauchinone **127,** and tabersonine **128** were recorded from Autodock Vina. Protein–ligand interaction results have shown that palmatine **126**, sauchinone **127**, and tabersonine **128** (Figure 9) were bound to the active site of the M^pro^. Further, MD simulation studies were performed, and the MM/PBSA results reflected that two compounds, palmatine **126** and sauchinone **127,** formed very stable complexes with the M^pro^ and showed free energy values of −71.47 kJ/mol and −71.68 kJ/mol, respectively, compared to the reference (−69.58 kJ/mol) [105]. Bharadwaj et al. (2021) extracted some natural compounds from *Echinacea angustifolia* and docked them against the main protease of a novel coronavirus. Almost 50 natural compounds showed binding affinities ranging from −12.93 to 0.0897 kcal/mol, and top five compounds (echinacoside **129**, quercetagetin 7-glucoside **130**, levan N **131**, inulin from chicory **132** (Figure 9), and 1,3-dicaffeoylquinic acid **133**) were selected for further analysis. Redocking, intermolecular interaction, and MD simulation studies showed that all five selected compounds exhibited a binding affinity of >10 kcal/mol [106]. Papia Chowdhury in 2021 screened the chemical constituents of *Tinospora cordifolia* (an Indian medicinal plant) and showed that berberine **134** (Figure 10), choline **75**, and tetrahydropalmatine satisfied all the required screening attributes. Molecular docking and MD simulation studies showed that among all the tested compounds, berberine **134** exhibited strong binding affinity and better inhibition towards the 3CL^pro^. All the inhibitors possessed drug-like properties and better pharmacokinetics [107] (Table 2).

Shree et al. (2022) investigated the potency of various natural compounds from three different plant species, including *Withania somnifera*, *Tinospora cordifolia*, and *Ocimum sanctum*, against the M^pro^ of SARS-CoV-2. Six phytochemicals showed better binding results and stable complexes with the target, i.e., withanoside V **43**: somniferine **135**, tinocordiside **136**, vicenin **137**, 4′-*O*-glucoside 2″-*O*-p-hydroxybenzoate **138**, and ursolic acid **139** (Figure 10). Furthermore, ADMET and drug-likeness analysis showed decent results [108]. Four active compounds that included three flavonoids (podocarpus flavone A, methoxyquercitrin, and proanthocyanidin) and a lignoid, chimarrhinin **140**, displayed the best inhibitory potential against the main protease. Among these four, the lignoid **(140)** showed the highest binding affinity (−9.0 kcal/mol) compared to the reference (−8.9 kcal/mol) [109]. The binding capacity of five naturally occurring alkaloids—berberine **134**, lycorine **141**, hemanthamine **142**, aloperin, and dendrobine—to SARS-CoV-2’s main protease was monitored. The molecular docking results revealed that Compounds **141** and **142** showed the best docking scores. In addition, pharmacokinetics and ADMET analysis confirmed the biocompatibility and drug-like properties of the tested compounds, especially **141** and **142** [110]. Laxman Durgam and Lalitha Guruprasad recently performed a virtual screening of natural compounds from the NCI database. Using AutoDock Vina and CDOCKER, eight active compounds that showed the best docking scores in the range of –7.3 kcal/mol to –8.1 kcal/mol were selected, and after assessing their drug-likeness properties, molecular dynamic stimulations were performed. The amino acid residues that highly contributed to the binding free energies of all the compounds were Cys145, Met165, and Glu166. The binding affinities of Compounds **143, 144, 145**, and **146** (Figure 10) were recorded. The complex of Compound **143** with the targeted enzyme was found to possess high conformational changes [111]. (The docking scores and amino acid residue interactions are depicted in Table 2).

Hawary et al. (2022) identified the active metabolites of *Citrus nobilis* L. and *Citrus deliciosa Tenora*. It was found that out of 21 compounds that belong to coumarins, phenolic acids, and flavonoids, quercetin-7-*O*-glucoside-3-*O*-rutinoside **147** showed the best docking score towards the active site of the main protease, followed by luteoline-7-rutinoside **148**, quercetin-3-*O*-rutinoside **149**, and apigenin-8-*C*-glucoside **150** (Figure 10). MD simulation studies revealed that Compounds **148** and **149** showed better binding with the active site of the M^pro^ [112]. Out of these 100 screened metabolites, pyranonigrin A **151** exhibited similar behavior as the reference compound and showed the best docking score of −7.3 kcal/mol. The MD simulation studies showed similar behaviors of both ligands (N3 and pyranonigrin A **151**) towards binding with the M^pro^, and the ADMET analysis results were found to be acceptable [113]. The phytochemicals of Indian medicinal plants showed 26 active compounds against SARS-CoV-2. Among these, the compounds 4,8-dihydroxysesamin **152** and arboreal **153** showed the best activities against PL^pro^ and 3CL^pro^, respectively. Molecular docking studies revealed that 4,8-dihydroxysesamin **152** showed binding affinity with PL^pro^, and arboreal **153** (Figure 10) showed binding affinity with 3CL^pro^ [114] (Table 2).

Moharana et al. (2022) performed an in silico analysis on 12 biologically active compounds out of 424 that were isolated from the extract of medicinal plants. It was shown that acacetin **154** (Figure 10) showed the best docking results and binding interactions with various amino acid residues of the SARS-CoV-2 main protease. MD simulation studies and free energy calculation analyses confirmed the flexibility and stability of the ligand–receptor protein complex [115]. Epoxy-linalool oxide **155** that was found in *Cymbopogon citrates* oil showed good binding interactions with the main protease. MD stimulation and pharmacokinetic studies revealed that epoxy-linalool oxide **155** possessed a more stable complex with the target protein and had more drug-likeness properties [116]. The potential of glycyrrhizin **156** towards M^pro^ and PL^pro^ inhibition showed good docking scores [117]. An active compound, rutin **29**, was isolated from a library of phytochemicals from Peruvian plants. MM/GBSA analysis of Compound **29** showed favorable interactions (−40.293 kcal/mol and 21.713 kcal/mol) with the M^pro^ and PL^pro^, respectively [118]. Africa et al. (2022) found some potent antitubercular phytochemicals and analyzed their potential for novel coronaviruses. Vobtusine lactone **157** showed greater binding affinity with 3CL^pro^, and deoxyvobtusine lactone **158** showed higher binding affinity with PL^pro^. ADMET analysis confirmed the drug-likeness attributes of these active compounds [119]. Some natural compounds with antiviral activities were selected and docked against the main protease. Among the tested compounds, seven compounds were found to be more potent based on their high binding affinities with target proteins. The highest binding affinity was reported for sotetsuflavone **159** (Figure 10) with hydrogen bonds and other alkyl interactions with various residues. ADMET analysis and pharmacokinetic studies showed good results for this compound, confirming its ability to act as an inhibitor of SARS-CoV-2 [120]. (Binding affinities and amino acid residue interactions are mentioned in Table 2).

Shaldam et al. (2021) screened many phenolic and terpene compounds from honeybee products against SARS-CoV-2 protein targets using in silico techniques. Through molecular docking analysis, it was found that Compounds **160**, **23**, **161**, **162**, and **163** (Figure 10) were more potent against the main protease of virus [121]. Maackin A **164** [91], anthracene dione **165** [92], and fortunellin **166** [93] were investigated as the M^pro^ inhibitors, and jezonofol **167** was identified as a PL^pro^ inhibitor.

The antiviral potential of the secondary metabolites of *Streptomyces* sp. GMR22 was evaluated by Melinda et al. (2021). Two active compounds, echoside A **168** and echoside B **169**, displayed a higher docking score than remdesivir towards 3CL^pro^ [122]. Suleimen et al. (2022) reported the anti-SARS-CoV-2 PL^pro^ inhibition potential of the two active compounds 6-demethoxy-4′-*O*-capillarsine **170** and tenuflorin C **171** from *Artemisia commutata* and *A. glauca*, respectively. The results from molecular docking analysis demonstrated that Compounds **170** and **171** (Figure 10) showed a good binding score and had interactions with various residues [125]. Similarly, Dutta et al. (2021) analyzed the antiviral potential of a medicinal plant, *Calotropis gigantean*, towards SARS-CoV-2 M^pro^ inhibition via in silico approaches. Among various bioactive compounds, juniper camphor **172** (Figure 10) displayed the best docking score [123] (Table 2).

The active site of an enzyme further comprised two sites: the binding site and the catalytic site [150]. Catalytic site residues reported in the literature are for the M^pro^, HIS41 and CYS145 [75]; for PL^pro^, HIS272, ASP28 and TRP106 [89]; for RdRp, SER759, ASP760, and ASP761 [77,89]; and for endoribonuclease, HIS235, HIS250, LYS290 [145], and THR341 [85]. The catalytic residues are shown in blue, and the binding site residues are shown in black in the following table.

### 5.2. Natural Compounds as SARS-CoV-2 Spike Protein Inhibitors

Glycosylated spike proteins that are present on the outer surface of the viral membrane are responsible for the attachment and entry of viruses into the host cell. The host angiotensin-converting enzyme 2 (ACE2) receptor and GRP78 binding domain are responsible for viral attachment and entry. Inhibition of spike protein binding is an alternate route to achieve viral inhibition. Different researchers have performed in silico studies on natural compounds and the spike protein of SARS-CoV-2.

**Figure 11:**
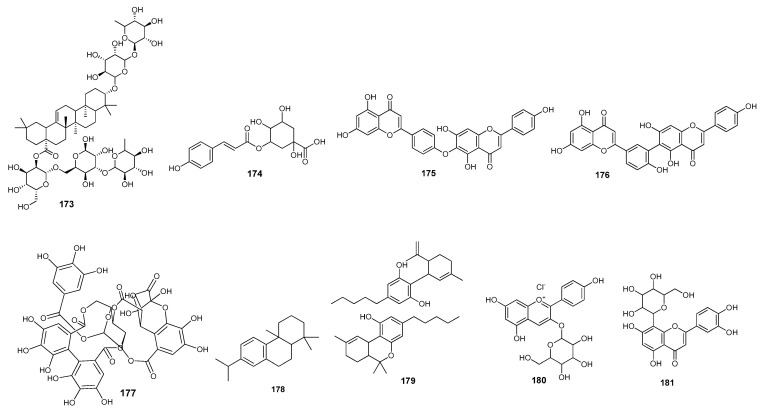

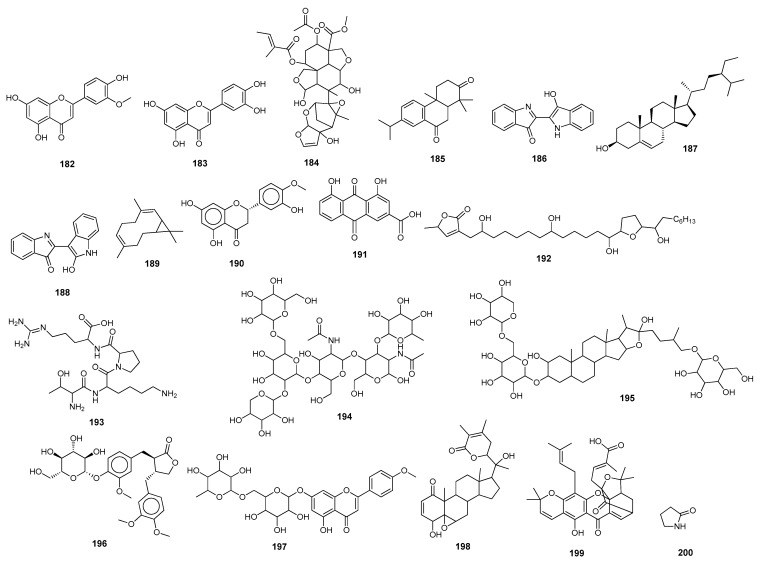
Natural products as spike protein inhibitors.

Muhseen et al. (2020) demonstrated terpenes are SARS-CoV-2 spike receptor binding domain attachment inhibitors to the human angiotensin-converting enzyme 2 (*h*ACE2) receptor via molecular docking, ADMET screening, and MD simulations. A terpene, NPACT01552 **173** (Figure 11), was discovered to be more potent than the others [126]. Potential phyto compounds of *Brassica oleracea* and naturally occurring biflavones were identified as the targets of the spike protein of SARS-CoV-2 through molecular dynamics, classical molecular dynamics simulations, and ADMET analysis. 3-*p*-coumaroylquinic acid **174** had a high affinity for the S2 domain of SARS-CoV-2 spike proteins [138]. Hinokiflavone **175** and robustaflavone **176** were found to be more active than nefamostat, which had a binding energy value of −8.40 kcal/mol [127]. Geraniin **177** was found to be an effective blocker of the interaction between the spike protein receptor binding domain and the human ACE2 receptor. The simulation demonstrated that geraniin **177** might bind more steadily to the spike protein than to the *h*ACE2 receptor [132]. Abietatriene **178** (Figure 11) was demonstrated as the best active inhibitor of spike proteins [128]. Quercetin **40** was found to be potent enough to block interaction sites on spike proteins [77]. Hesperidin **51** and nabiximols **179** (Figure 11) performed inhibitory activity against spike proteins with binding free energy values compared to the standard drugs nelfinavir, hydroxychloroquine sulphate, and chloroquine [86]. Nine molecules from Djiboutian medicinal plants were studied, out of which rutin **29**, catechin **41**, and kaempferol **23** exhibited lower binding energy values than the reference drug, hydroxychloroquine (−4.828 kcal/mol) against the SARS-CoV-2 receptor binding domain [69]. Amentoflavone **42** was found to be the best active compound against the spike proteins compared to the reference drug hydroxychloroquine, with a binding free energy of −6.4 kcal/mol. Lipinski’s rule of five also showed its drug-likeness [78]. Crocin **(63)** was shown to be an active carotenoid against the spike protein of SARS-CoV-2 [73].

Mehmood, A. et al. (2021) examined Quranic and prophetic medicinal plants as inhibitors of the SARS-CoV-2 essential enzyme spike protein. These inhibitors showed a strong binding affinity for pelargonidin-3-galactoside **180** (Figure 11) [90]. Bhowmik et al. (2020) identified various phytochemicals that showed binding with the SARS-CoV-2 spike protein and with the GRP78 binding domain. The molecular docking results of the tested phytochemicals showed that orientin **181** (a flavonoid) showed the best binding affinity with the SARS-CoV-2 spike protein and with the GRP78 binding domain. It was also found that orientin **181** showed binding interactions with spike proteins in the same region where GRP78 interactions occurred. Further, MD simulation studies confirmed that orientin formed stable complexes with the GRP78 binding domain and inhibited the attachment of the SARS-CoV-2 spike protein to this receptor [129]. A total of 37 compounds of *Kabasura Kudineer*, Official Siddha Formulation, and JACOM were screened. It was shown that chrysoeriol **182**, luteolin **183**, quercetin **40**, and scutellarein **118**, possessed high binding affinities by forming hydrogen bonds with four amino acid residues of the target protein. Pharmacokinetic analysis showed that all the selected compounds possessed good bioavailability and no toxicity [130]. Acacetin **154** showed the best docking result with a binding energy of −7.75 kcal/mol by forming hydrogen bonds and hydrophobic interactions with various residues. MD simulation studies and free energy calculation analyses confirmed the flexibility and stability of the ligand–receptor protein complex [115]. Mhatre et al. (2021) investigated the therapeutic potential of different active catechins against SARS-CoV-2 spike proteins using computation techniques. Molecular docking studies revealed that all the tested catechins showed better docking scores in the range of −6.3 kcal/mol to −5.7 kcal/mol than the reference NGA (−5.0 kcal/mol). Among all the screened compounds, epigallocatechin gallate **21** exhibited the highest docking score, and MD simulation studies confirmed the stability of the protein–ligand complex. The pharmacokinetic analysis results revealed that the drug-likeness of these compounds needs to be improved [131]. The neem plant extract was evaluated for its inhibition of SARS-CoV-2 entry into the host cell. Out of the total 19 compounds that were extracted and screened, 3 compounds demonstrated the best binding scores towards the spike receptor binding domain–angiotensin-converting enzyme 2 complex (RBD–ACE2) compared to the reference, hesperidin **51** (−7 kcal/mol). Azadirachtin H **184** showed a higher binding affinity than quercetin **40** and margocin **185**. Compound **184** also showed better binding interactions (−8 kcal/mol) with spike proteins and the ACE2 receptor. The MM/PBSA binding free energy calculations and pharmacokinetic studies confirmed the potential of the studied compounds as drug candidates towards spike SARS-CoV-2 inhibition [139]. (The binding energies and amino acid residue interactions are described in Table 2).

Cheke et al. (2021) analyzed the potential of various medicinally active phytochemicals towards spike protein–ACE2 inhibition. Molecular docking studies revealed that among various screened compounds, the higher binding affinity was shown by indigo blue **186**, followed by glycyrrhizin **156**, β-sitosterol **187**, indirubin **188**, bicyclogermacrene **189**, curcumin **86**, hesperetin **190**, rhein **191**, and berberine **134** [140]. Acetogenins isolated from *A. muricate* showed good docking scores with the target protein in the range of −5.3 to −7.7 kcal/mol. The highest binding affinity was reported for *cis*-annonacin **192** compared to the reference (−7.5 kcal/mol) [133]. Tuftsin **193**, a naturally occurring peptide, possessed binding interactions with both ACE2 and neuropilin-1 (NRP1), with binding affinities of −6.9 kcal/mol and −8.1 kcal/mol, respectively. The surface plasmon resonance (SPR) analysis further proved the potency of tuftsin towards the inhibition of SARS-CoV-2 spread [141]. Thuy et al. (2021) evaluated 34 compounds that were found in *Cymbopogon citratus* oil against ACE2 receptor proteins using docking and MD simulation studies. It was found that five compounds showed the best binding affinities, of which the highest affinity was shown by epoxy-linalool oxide **155** through hydrogen bonding and van der Waals interactions with different residues. MD stimulation and pharmacokinetic studies revealed that Compound **155** possessed a more stable complex with the target protein and had a more drug-like attitude [116]. Bromelain **194** (Figure 11) (an enzyme, present in fruits) bound more effectively in the region of RBD-ACE2 binding sites. The molecular docking results of bromelain with the receptor binding domain (RBD) variants, WT, the United Kingdom, BR, SA, and the United States, possessed binding affinities of −14.9 kcal/mol, −15.0 kcal/mol, −15.6 kcal/mol, −15.4 kcal/mol, and −15.0 kcal/mol, respectively. MD simulation studies were also performed to confirm the stability of these complexes [151]. Dharmashekara et al. (2021) conducted in silico investigations on various phytochemicals to evaluate their potential towards the spike proteins of SARS-CoV-2. Among the tested compounds, trigoneoside IB **195** showed the highest binding affinity with target spike glycoproteins, with interactions with various amino acid residues [134]. Two active compounds, **196** and **197**, extracted from Jinhua Qinggan granules (JQGs), were analyzed against ACE2. Arctiin **196** and linarin **197** (Figure 11) showed the best docking scores, having strong interactions with METB1007 and ALA1000 amino acid residues, respectively [135] (Table 2).

Kashyap et al. (2021) investigated the anti-COVID-19 activity of phytochemicals from some medicinal plants. The isolated compounds were docked against spike proteins of the virus and two host proteins (ACE2 and TMPRSS2). Out of 12 screened compounds that showed the best binding affinities with target proteins, the highest docking score was shown by withanolide D **198** (Figure 11) with the spike proteins, ACE2, and TMPRSS2, respectively. ADMET analysis confirmed the drug-likeness of these compounds [136]. Some natural compounds with antiviral activities were tested against spike glycoproteins and ACE2. Among the tested compounds, seven compounds were found to be more potent based on their high binding affinities with the target proteins. The highest binding affinities were reported by morellic acid **199** against spike proteins and human ACE2, respectively [120]. Roshni et al. (2022) investigated the phytochemicals of Indian medicinal plants and reported 26 active compounds against SARS-CoV-2. Among these, scutellarein **118** showed the best activity against the spike glycoproteins of SARS-CoV-2 [114]. Melinda et al. (2021) evaluated the antiviral activity of metabolites of *Streptomyces* sp. GMR22 against spike proteins and ACE2 receptors. Echoside A **168** and echoside B **169** displayed higher docking scores than remdesivir against the target proteins [122]. Siddiqui et al. (2022) analyzed the therapeutic potential of an ethanolic extract of *Moringa oleifera* fruits against SARS-CoV-2. Among all the extracted phytochemicals, 2-pyrrolidinone **200** (Figure 11) displayed good binding interactions with both the spike protein and the ACE2 receptor [137].

### 5.3. Natural Compounds as SARS-CoV-2 RdRp Inhibitors

RNA-dependent RNA polymerase (RdRp) is involved in viral RNA replication and transcription. Various researchers have found some active natural phytochemicals against this enzyme.

**Figure 12:**
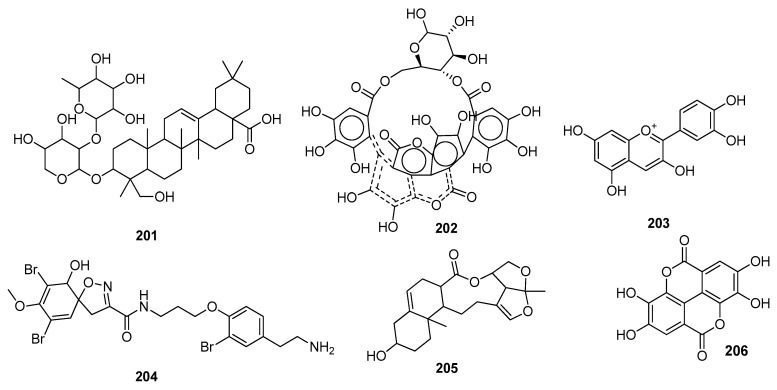

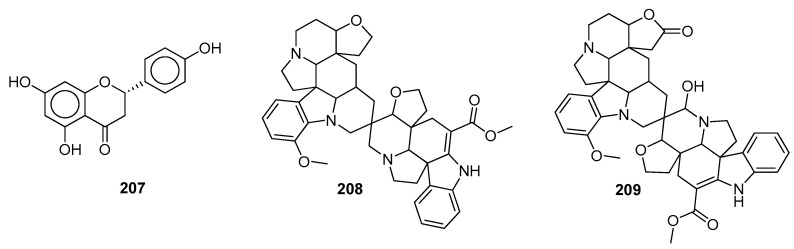
Natural products as inhibitors of replication enzyme (RdRp) of SARS-CoV-2.

Mir, S.A. et al. (2021) examined SARS-CoV-2 RdRp potential inhibitors extracted from *Nigella sativa* through an in silico approach. When compared to the standard inhibitor remdesivir, α-hederin **201** (Figure 12) displayed the highest binding affinity to RdRp (PDB ID: 6M71) [142]. Some Quranic and prophetic medicinal plants acted as potential inhibitors of the SARS-CoV-2 essential enzymatic functions of RdRp; kaempferol **23** inhibited the viral transcription machinery in the best way [90]. Polyphenolic anti-HIV reverse transcriptase natural compounds were reported as potential inhibitors of NSP12 (RdRp) of the SARS-CoV-2. RNA-dependent RNA polymerase (RdRp) polymerizes the nucleotide chain of the daughter strand, which helps in the inhibition of virus proteins. NSP12 (RdRp) demonstrated a good binding score with ellagitannin punicalin **202** [79]. Cyanidin **203** was found to be active with a −7.7 kcal/mol binding affinity value against RdRp [77]. Amphimedoside C **78** was a potent inhibitor of SARS-CoV-2 RdRp [87]. 14-debromoaraplysilin I **204** [88], glaucogenin D **81**, glaucogenin C **205** (Figure 12) [89], crocin **63**, a food-derived carotenoid [73], and jezonofol **167** [91] were demonstrated to be the best active compounds against the replication enzyme RdRp (as depicted in Table 2).

Shaldam et al. (2021) screened many phenolic and terpene compounds from honeybee products. Through molecular docking analysis, it was found that ellagic acid **206**, kaempferol **23**, quercetin **40**, *p*-Coumaric acid **163**, and naringenin **207** were more potent against the RdRp enzyme of SARS-CoV-2. Compound **206** showed hydrogen bond interactions with RdRp active site amino acid residues [121]. Some potent antitubercular phytochemicals were analyzed against the novel coronavirus. It was demonstrated that 10 compounds showed active interactions with various protein targets of the virus. Among these, vobtusine lactone **157**, deoxyvobtusine lactone **158**, deoxyvobtusine **208**, and globospiramine **209** showed greater binding affinities with the RdRp of SARS-CoV-2. The most potent compound, **157**, showed many interactions with active site residues, including hydrogen bond interactions with SER682 and ALA688 residues. ADMET analysis confirmed the drug-likeness attributes of these active compounds [119]. Roshni et al. (2022) investigated the phytochemicals of Indian medicinal plants and found 26 active compounds against SARS-CoV-2. Among these, the compound arboreol **153** showed the best activity against the RdRp proteins of the virus [114]. Melinda et al. (2021) displayed the antiviral potential of two active compounds named echoside A **168** and echoside B **169**. Both displayed higher docking scores than remdesivir against the target proteins of SARS-CoV-2 [122] (Table 2).

### 5.4. Natural Compounds as SARS-CoV-2 Nucleocapsid Inhibitors

**Figure 13:**
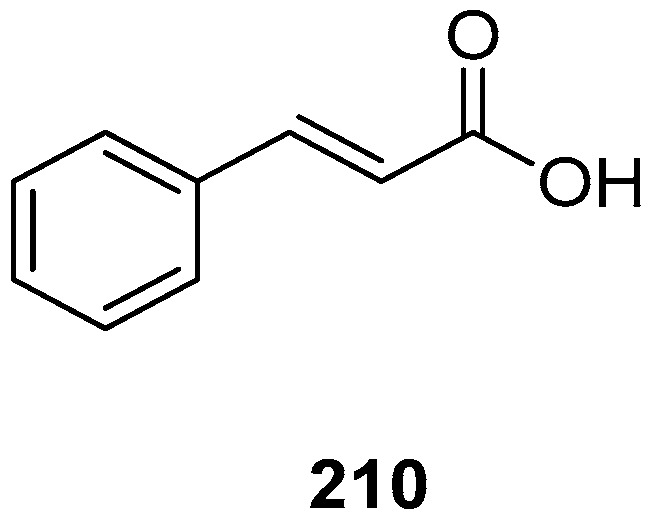
Natural compound as nucleocapsid inhibitor of SARS-CoV-2.

Nucleocapsids or *N*-proteins are important structural and functional proteins that have been found to play an important role in viral replication and translational properties. They have two domains, i.e., NTD (N-terminal domain) and CTD (C-terminal domain), both of which function to bind with viral RNA and translate it. So, the inhibition of *N*-proteins is considered an important target to control this viral disease. Roshni et al. (2022) investigated the phytochemicals of Indian medicinal plants and found 26 active compounds against SARS-CoV-2. Among these, 4,8-dihydroxysesamin **152** and arboreol **153** showed the best activities against the nucleocapsid proteins of SARS-CoV-2. Molecular docking studies revealed that Compounds **152** and **153** showed the highest binding affinities. Both of these compounds were found to be efficient compared to the reference drugs [114]. It was found that glycyrrhizin **156** showed the highest binding affinity with nucleocapsid proteins. The MM/GBSA and MM/PBSA estimations showed binding free energies of −30.05 kcal/mol and −25.95 kcal/mol, respectively, for the ligand–receptor complex [117]. Mamani et al. (2021) found active rutin **29** from a library of phytochemicals from *Peruvian* plants. MM/GBSA analysis of the compound showed favorable interactions (−34.342 kcal/mol) with the *N*-domain of the nucleocapsid, and further MD simulation studies confirmed the stability of the ligand–receptor complex [118]. It was shown that trigoneoside IB **195** exhibited the highest binding affinity, with target proteins having interactions with different amino acid residues. Other than this, two more compounds showed docking scores of −4.8 kcal/mol and −4.6 kcal/mol, respectively [134]. Husain et al. (2022) analyzed the antiviral effect of some bioactive compounds against the NTD and CTD of the nucleocapsid proteins of SARS-CoV-2. The compounds were isolated using HPLC, and then docking studies were performed, followed by MD simulation and pharmacokinetic analysis. From the molecular docking results, it was found that with the NTD, apigenin **120** showed the highest docking score, followed by catechin **41** and apiin **53**. With the CTD of the nucleocapsid, apigenin **120** showed the highest score, followed by cinnamic acid **210** (Figure 13) and apiin **53**. MD simulation and pharmacokinetics confirmed the therapeutic potential of these four compounds towards the inhibition of this viral disease [143]. The inhibitory potential of curcumin **86** towards SARS-CoV-2 nucleocapsid proteins was evaluated. Molecular docking and MD simulation analyses were carried out to find the binding affinity of the tested ligand with the target proteins. The study concluded that curcumin formed a stable complex with the target protein and would prove the best candidate for SARS-CoV-2 drug development [144]. (The docking scores and amino acid residue interactions are listed in Table 2).

### 5.5. Natural Compounds as SARS-CoV-2 Endoribonuclease Inhibitors

**Figure 14:**
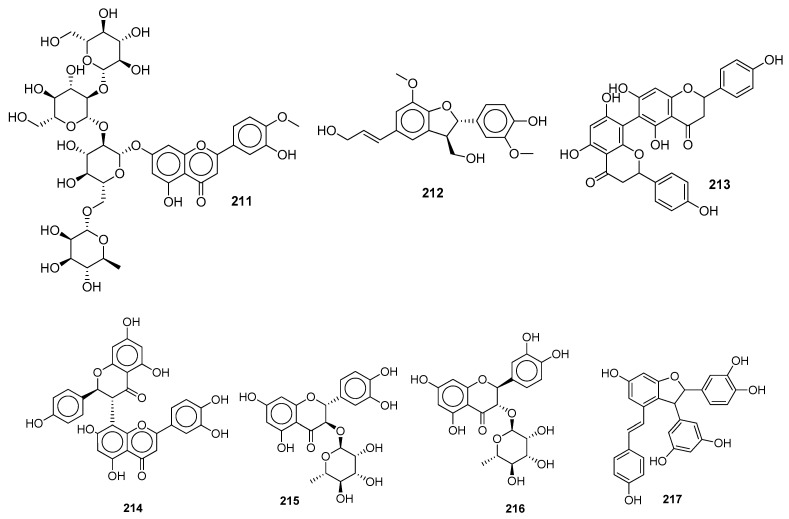
Structures of the SARS-CoV-2 helicase inhibitors.

Endoribonucleases prevent host dsRNA sensors from recognizing dsRNA intermediates. The biflavonoid hinokiflavone **175** [79] and rutin **29** [85] exhibited a good binding score against NSP15.The diosmetin glucopyranoside derivative NPC198199 **211** [145] and the NPASS compound with the ID NPC10737 **212** (Figure 14) [146] both demonstrated the greatest anti-NSP15 potency.

### 5.6. Natural Compounds as SARS-CoV-2 Helicase Inhibitors

Crocin **63** [73], the natural product biflavonoid rhusflavanone **213**, morelloflavone **214** [79], and scirpusin A **217** (Figure 14) [91] have the highest binding affinities towards helicase (essential for replication). NPASS compounds with the IDs NPC270578 **215** and NPC52382 **216** (Figure 14) both displayed a higher MMGBSA score [146].

### 5.7. Natural Compounds as SARS-CoV-2 Methyltransferase Inhibitors

**Figure 15:**
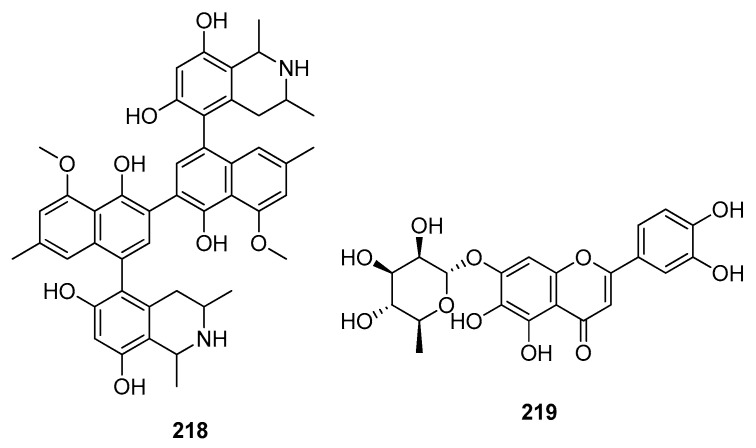
Natural compounds as SARS-CoV-2 anti-methyltransferase agents.

Polyphenolic compounds, which were well known as anti-HIV reverse transcriptase inhibitors, also played a role as potential inhibitors of the nonstructural protein NSP16–NSP10 complex (*S*-adenosylmethionine complex) of SARS-CoV-2. In the case of SAM-dependent 2′-*O*-methyltransferase complex enzymes (NSP16–NSP10 complex), the biflavonoid robustaflavone **176** and the alkaloid michellamine B **218** (Figure 15) both demonstrated active binding affinities [79]. The NPASS compound with the ID NPC226294 **219** (Figure 15) exhibited good binding free energies towards methyltransferase with an MM/GBSA score of −75.24 compared to the control, i.e., sinefungin, with an MM/GBSA score of −60.32 [146].

### 5.8. Natural Compounds as SARS-CoV-2 ADP-Ribose Phosphatase Inhibitors

The NSP–complex formation was potentially blocked by ADRP inhibitors. In this context, Mujwar, S. et al. (2022) investigated food-derived carotenoids against the ADP-ribose phosphatase (ADPRP) (PDB ID: 6W02) of SARS-CoV-2 through an in silico approach. Crocin **63** was also reported as a potential inhibitor of ADPRP [73].

### 5.9. Natural Compounds as SARS-CoV-2 Exoribonuclease Inhibitors

**Figure 16:**
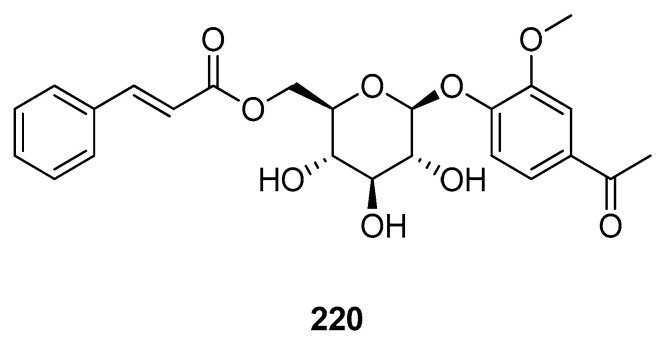
Exoribonuclease inhibitor of SARS-CoV-2.

An exoribonuclease inhibitor was identified by Naik et al. (2020). Naik and co-workers tackled the bioactive compounds from the Natural Product Activity and Species Source database that might impede the activity of the essential enzyme of SARS-CoV-2, i.e., exoribonuclease, through a molecular docking study. It was observed that the NPASS compound with the ID NPC137813 **220** (Figure 16) displayed the highest binding capability to exoribonuclease compared to the control MES, with a −32.10 binding score [146]. (The binding affinities and amino acid residue interactions are represented in Table 2).

### 5.10. Natural Compounds as Inhibitors of Other SARS-CoV-2 NSPs

**Figure 17:**
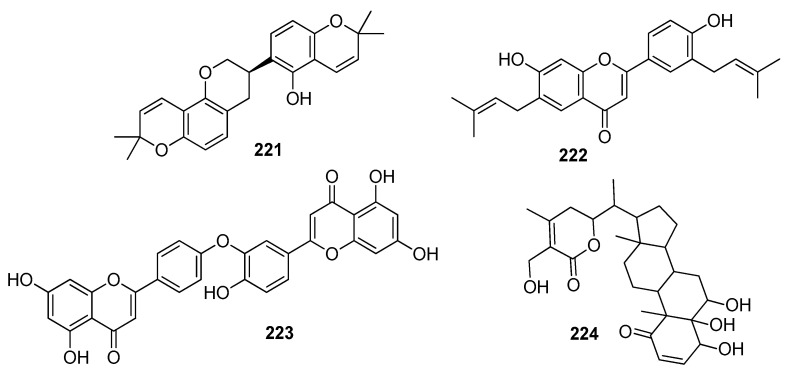
Natural products acting as SARS-CoV-2 NSP inhibitors.

Medicinal plant metabolites acted as potent inhibitors of the SARS-CoV-2 NSP9 RNA-binding protein. Those inhibitors were identified by Bandyopadhyay, S. et al. (2021) through a molecular dynamics evaluation. Hispaglabridin B **221**, licoflavone B **222**, and ochnaflavone **223** (Figure 17) were found to have the best binding with the SARS-CoV-2 NSP9 protein [147]. 2,3-dehydrosomnifericin **224** was also identified as an NSP3 inhibitor [148].

### 5.11. Natural Compounds as SARS-CoV-2 Envelope Protein Inhibitors

**Figure 18:**
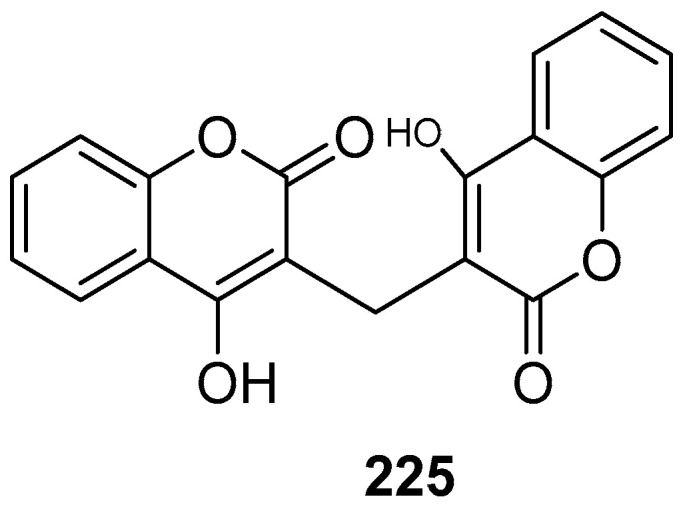
Envelope protein inhibitor of SARS-CoV-2.

Coronavirus E proteins are incorporated into the virion lipidic envelope along with the spike protein (S) and the membrane protein (M). Roshni et al. (2022) investigated the phytochemicals of Indian medicinal plants and found 26 active compounds against the SARS-CoV-2 envelope protein. Among these, dicumarol **225** (Figure 18) showed the best activity against envelope protein. Molecular docking studies revealed that Compound **225** showed a binding affinity of −7.4 kcal/mol against the target protein [114]. Similarly, trigoneoside IB **195** showed the highest binding affinity against target *E*-proteins with interactions with various amino acid residues [134] (Table 2).

In this review, we have highlighted the potent anti-SARS-CoV-2 activities of natural compounds that constitute various drug classes, i.e., flavonoids, bioflavonoids, alkaloids, carotenoids, terpenes, steroids, quinones, polyphenols, and glycosides. Numerous docking simulations studies have recommended to use these compounds as COVID-19 therapy. These classes have shown their promising actions on multiple therapeutic targets of SARS-CoV-2. Among them, flavonoids and their subgroups have shown their potential in inhibiting the viral infection by targeting all the enzyme targets of SARS-CoV-2. For example, amentoflavone **42,** one of the most abundant plant flavonoids, is proposed as a lead candidate with its ability to inhibit spike glycoproteins, viral proteases (M^pro^ and PL^pro^), and RdRp activities of the virus, as well as to inhibit the ACE2 activity of the host cell [61,78,79]. Rutin **29**, which is a flavonoid glycoside, showed inhibitory effects on the main protease, PL^pro^, RBD-ACE2 complex, nucleocapsid, and endoribonuclease [68,69,85,118]. Apart from this, many other flavonoids (myricetin **33** [54,55], baicalein **16** [31,32], kaempferol **23** [54,57], quercetin **40** [54,60], and catechins **41** [54,60,69]) have been found to display encouraging in silico outcomes against the COVID-19 disease. It was also found that this class of compounds has shown interactions with the catalytic and binding site amino acid residues of targeted enzymes.

Moreover, carotenoids, such as crocin **63**, showed potent anticoronavirus properties by inhibiting the coronavirus targets (the M^pro^, spike glycoproteins, RdRp, helicases, and ADP-phosphates) [73]. The other drug classes, i.e., alkaloids (tubocuraine **74** [83] and palmatine **126** [105]), terpenes (cyanopyrin **49** [66], ferolide **68** [80], and abietatriene **178** [128]), steroids (withanolide R **50** [67], withacoagulin H **60** [72], withacoagulin **62** [72], ajugin E **61** [72], sterenin M **65** [75], and stigmasterol **77** [85]), quinones (pycnanthuquinone C **47** [65] and pycnanthuquinone B **48** [65]), polyphenols (geraniin **177** [132], ellagitannin punicalin **202** [79], and ellagic acid **206** [121]), and glycosides (cyanidin **203** [77], arctiin **196** [135], forsythiaside A **46** [64], and hesperidin **51** [86]) have also been found to show promising anti-SARS-CoV-2 activities. It is anticipated that the phytoconstituents discussed in this report will aid the development of an effective and safe anti-SARS-CoV-2 treatment option from naturally procured compounds.

## 6. Conclusions

The pandemic of the novel coronavirus disease has become challenging because of the lack of specific treatment and the continuous resistance caused by mutant strains of the virus. Chloroquine, hydroxychloroquine, ivermectin, azithromycin, remdesivir, lopinavir, ritonavir, favipiravir, galidesivir, dexamethasone, and ruxolitinib are considered the alternative treatments for this viral pandemic, but they are not as effective as one would hope. Natural products have been proven to be the best source of treatment for various human illnesses. In this regard, several plant-based remedies have been applied to alleviate COVID-19. This review article mainly focuses on all the natural compound-based treatments that have been suggested for SARS-CoV-2 through in vivo, in vitro, and in silico analyses. Many important phytochemicals, such as flavonoids, bioflavonoids, catechins, alkaloids, chalcones, terpenes, sterols, quinones, glycosides, and polyphenols, extracted from medicinal plants, algae, fungi, bacteria, and marine natural sources are suggested to be active ingredients to combat the coronavirus disease. Among them, flavonoids and their subgroups have shown their potential in inhibiting viral infection by targeting all the enzyme targets of the SARS-CoV-2. The main targets of these active natural compounds are the main proteases (M^pro^/3Cl^pro^), papain-like proteases (PL^pro^), viral spike glycoproteins, human receptor cells (ACE2, TMPRSS2, and NRP1), RBD-ACE2, RNA-dependent RNA polymerase (RdRp), nucleocapsid, endoribonuclease, helicase, methyltransferase (NSP16–NSP10 complex), exoribonuclease, nonstructural proteins, and envelope proteins.

Computational approaches are emerging techniques to analyze the potential of biologically active compounds against targeted diseases. These techniques help researchers find effective potential treatments against the novel SARS-CoV-2. In recent years, various in silico analyses, such as molecular docking, MD simulations, MM/GBSA, MM/PBSA, ADMET, and Lipinski’s rule of five have been carried out to check the potential of active phyto-constituents towards targeted enzymes of this viral infection. Many active natural materials with greater binding affinities towards targeted areas are highlighted in this review article. Moreover, it is suggested that the antiviral benefits of these natural compounds should be studied on an experimental level, which will benefit the researchers in designing new anti-SARS-CoV-2 drugs.

## Figures and Tables

**Table 2:** In silico results for compounds for which no in vitro studies are available.

Serial No.	Compound Name	Binding Potential		Interactions at the Enzyme Active Site *		References
		Docking Score	Binding Free Energy	H-Bond Interactions	Hydrophobic and Other Interactions	
**Major Protease (M^pro^/3CL^pro^)**						
**1**	Forsythiaside A **(46)**	−8.08 kcal/mol	−121 ± 19 kJ/mol	THR190, PRO168	**HIS41**, ASP187	[64]
**2**	Pycnanthuquinone C **(47)**	-	−7.8 kcal/mol	**HIS41**	GLU166, HIS164, **CYS145**, LEU27	[65]
**3**	Pycnanthuquinone B **(48)**	-	−8.3 kcal/mol	GLY143, SER144	**HIS41**, HIS164	[65]
**4**	Cyanopyrin **(49)**	−4.78 kcal/mol	−7.4 kcal/mol	THR111, ASP153, SER158	PHE294, ILE106	[66]
**5**	Withanolide R **(50)**	−9.63 kcal/mol	−141.96 kJ/mol	GLN189, **HIS41**	MET165, PRO168, ASP187, ALA191	[67]
**6**	Hesperidin **(51)**	−178.5910 kJ/mol	-	GLU166, GLN192, ARG 188, GLN189, MET49, ASP187, TYR54, LEU141, SER144, HIS163, THR26	-	[68]
**7**	Rutin **(29)**	−176.2740 kJ/mol −9.09 kcal/mol	- -	**CYS145**, ASN142, PHE140, GLU166, GLN192, THR190, ASP187, TYR54, HIS164, GLU166, LEU8	- -	[68] [69]
**8**	Diosmin **(52)**	−174.126 kJ/mol	-	GLN192, THR190, ARG188, HIS164, GLN189, GLU166, GLY143, SER144, **CYS145**	-	[68]
**9**	Apiin **(53)**	−171.008 kJ/mol	-	THR190, SER144, LEU141, HIS163, **CYS145**, THR26	-	[68]
**10**	Jaceidin **(54)**	−7.3 kcal/mol	-	LEU141, GLY143, SER144, **CYS145**, ARG188,	-	[70]
**11**	Pachypodol **(55)**	−7.1 kcal/mol	-	GLY143, SER144, **CYS145**	-	[70]
**12**	Chrysosplenetin **(56)**	−7.1 kcal/mol	-	LEU141, GLY143, SER144, **CYS145**	-	[70]
**13**	Imidazoline-4-one-2-imino-1-(4-methoxy-6-dimethylamino-1,3,5-triazin-2-yl) **(57)**	−7.013 kcal/mol	-	GLU166, GLN192	-	[71]
**14**	Spiro[4,5]dec-6-en-1-ol, 2,6,10,10-tetramethyl **(58)**	−6.369 kcal/mol	-	-	MET165, **HIS41**, MET49, **CYS145**	[71]
**15**	3-hydroxy-5-cholen-24-oic acid **(59)**	−6.251 kcal/mol	-	THR25, THR190, GLN192	MET49, **HIS41**, MET165	[71]
**16**	Withacoagulin H **(60)**	−11.1 kcal/mol	−63.463 kJ/mol	GLY143, ARG188, THR190, GLN192	**HIS41**, **CYS145**, THR25, THR26, LEU27, MET49, ASN142, SER144, HIS164, MET165, GLN166, GLN189	[72]
**17**	Ajugin E **(61)**	−11.5 kcal/mol	−56.14 kJ/mol	GLN192, THR190	**HIS41**, **CYS145**, THR25, MET49, ASN142, GLY143, HIS164, MET165, GLU166, PRO168, ARG188, GLN189	[72]
**18**	Withacoagulin **(62)**	−10.8 kcal/mol	−44.496 kJ/mol	THR190, GLN192, GLY143	**HIS41**, **CYS145**, THR25, THR26, LEU27, MET49, ASN142, SER144, HIS164, MET165, ARG188, GLN189	[72]
**19**	Crocin **(63)**	−8.0 kcal/mol	-	ALA116, SER123, SER139, PHE140, LEU141, ASN142, GLU166, HIS172, GLN189		[73]
**20**	Rhamnocitrin **(64)**	−7.83 kcal/mol	−49.53 kcal/mol	GLU166, THR190	**HIS41**, **CYS145**, CYS44, TYR54, PRO52, MET49, ASP187, ARG188, GLN189, ALA191, GLN192, PRO168, LEU167, MET165, HIS164	[74]
**21**	Sterenin M **(65)**	−8.431 kcal/mol	−49.57 kcal/mol	GLU166, PHE140, HIS163, GLY143, ASN142, THR26, **HIS41**	LEU141, HIS172, SER144, LEU27, THR25, THR24, CYS44, TYR54, ASP187, ARG188, MET49, GLN189, HIS164, MET165	[75]
**22**	3,4,5-tricaffeoylquinic acid **(66)**	−9.0 kcal/mol	-	THR190, ARG188, THR26, ASP187, GLY143, ASN142	MET165, MET49, PRO168, HIS172	[76]
**23**	Quercetin **(40)**	−9.2 kcal/mol	-	GLU290, GLU288, THR199	LYS137, ARG131, LEU272, TYR239, LEU287	[77]
**24**	Peonidin **(67)**	−8.4 kcal/mol	-	GLU290, ASP289, LYS5GLU288 LEU287, LEU271, GLY27, THR199, LEU272, GLU290		[77]
**25**	Catechin **(41)**	−7.67 kcal/mol	-	GLU166, HIE164	-	[69]
**26**	Kaempferol **(23)**	−7.215 kcal/mol	-	GLU166, GLN189, HIE164	-	[69]
**27**	Amentoflavone **(42)**	−9.8 kcal/mol −8.6 kcal/mol	- -	THR25, GLY143, GLN189, GLU166 CYS44, VAL186, ARG188, GLU166	MET49, **HIS41**, **CYS145**, THR24, THR45, MET165, TYR54, ARG188, ASP187, LEU167, PRO168, ASN142 THR25, **HIS41**, ASN142, **CYS145**	[78] [79]
**28**	Ferolide **(68)**	−7.9 kcal/mol	-	HIS163, **CYS145**, LEU141, ASN142	**CYS145**, **HIS41**, GLU166, SER144, PHE140, HIS164, MET165, GLN189, CYS144, MET49, THR25, GLY143	[80]
**29**	Ginkgolide A **(69)**	−65.412 kcal/mol	−63 kcal/mol	VAL72, LYS73, TYR135, GLY151, CYS144, **HIS41**		[81]
**30**	Gracillin **(70)**	−9.2 kcal/mol	-	GLU166, LEU141, SER144, **CYS145**, GLY143, ASN142	-	[82]
**31**	Proanthocyanidin **(71)**	−9.2 kcal/mol	-	GLU166, HIS164, HIS163, TYR54	**HIS41**, ASP187, MET165	[82]
**32**	Ginkgolide M **(72)**	−11.2 kcal/mol	-	ASN142, **CYS145**, GLU166, GLY143, HIS163, PHE140		[83]
**33**	Mezerein **(73)**	−11 kcal/mol	-	ASN142, **CYS145**, GLU166, GLY143, HIS163, HIS172, PHE140		[83]
**34**	Tubocuraine **(74)**	−10.9 kcal/mol	-	ASN142, **CYS145**, GLU166, GLY14		[83]
**35**	Choline **(75)**	−3.7 kcal/mol	-	GLY143, LEU141	ASN142, PHE140, HIS163, SER144, GLU166	[84]
**36**	Volkensiflavone **(76)**	−8.6 kcal/mol	-	**CYS145**, GLU166	THR25, **HIS41**	[79]
**37**	Stigmasterol **(77)**	−6.30 kcal/mol	-	PRO39	**CYS145**, LEU27, VAL42, **HIS41**	[85]
**38**	Hesperidin **(51)**	−8.3 kcal/mol	-	PHE140, GLU166, SER144, **CYS145**	MET49, LEU141, HIS163, THR26	[86]
**39**	Amphimedoside C **(78)**	-	−127.3 kJ/mol	ASN142, PHE140, LEU141, HIS163, SER144, **CYS145**	ASN142, HIS172, GLN189	[87]
**40**	Fasciospongide A **(79)**	-	−104.37 kJ/mol	**HIS41**, THR190, GLN192, VAL186	MET165, MET49, PRO168, ALA193, LEU167, PHE185, ARG188, VAL42, THR25, **CYS145**	[88]
**41**	Glaucogenin D **(81)**	−7.9 kcal/mol	-	GLY143, **HIS41**	MET165, THR24	[89]
**42**	Calcium elenolate **(83)**	−7.0 kcal/mol	-	GLU291	LYS152, PHE111	[90]
**43**	Maackin A **(164)**	−8.4 kcal/mol	−43.1 kcal/mol	THR26, **HIS41**, MET49, GLN192	THR25, GLY143, ASN142, MET165, PRO168	[91]
**44**	Anthracene dione **(165**)	−5.73 kcal/mol	-	GLY143	-	[92]
**45**	Fortunellin **(166)**	−13.9 kcal/mol	-	LEU32, ASP33, ASP34, VAL35, TYR37, GLN83, LYS88, TYR101, LYS102, PHE103, VAL104, ARG105, ASP108, PHE159, CYS160, ASP176, LEU177, GLU178		[93]
**46**	Curcumin **(86)**	−8.62 kcal/mol	-	THR26, GLY143, GLN189, THR190	-	[94]
**47**	Kolaviron **(87**)	−7.027 kcal/mol	-	GLU166, GLY143	-	
**48**	Bisdemethoxycurcumin **(88**)	−5.641 kcal/mol	-	THR26, THR190, GLN189, GLU166	-	[94]
**49**	6-gingerol **(89**)	−4.975 kcal/mol	-	THR190, GLN189	-	[94]
**50**	Artemisinin **(26**)	−4.252 kcal/mol	-	GLN 189, GLU 166	-	[94]
**51**	Luteolin **(90)**	−8.3 kcal/mol	-	GLN110, THR111 ASN151	-	[95]
**52**	Lucenin **(91)**	−10.7 kcal/mol	-	PHE219, LEU220, ARG222, ASN274	-	[95]
**53**	Olealonic acid **(92)**	−9.5 kcal/mol	-	ASP289	-	[95]
**54**	Isoorientin **(93)**	−9.2 kcal/mol	-	PRO52, TYR54, PHE181	-	[95]
**55**	Isochaphoside **(94)**	−10.5 kcal/mol	-	ARG222, ASN274, PHE219	-	[95]
**56**	Saponarin **(95)**	−10.6 kcal/mol	-	ARG40, PHE181	-	[95]
**57**	Schaftoside **(96)**	−10.2 kcal/mol	-	ASP197, ASN238, ARG131, LYS137	-	[95]
**58**	7,2″-bieckol **(98)**	−10.7855 kcal/mol	-	THR24, THR26, GLY143, GLU189	-	[96]
**59**	Sarelengan B **(105)**	−9.8 kcal/mol	-	**HIS41**, **CYS145**, GLU166	-	[97]
**60**	Bislatumlide A **(106)**	−9.6 kcal/mol	−34.8 kcal/mol	GLY143, GLU166	-	[97]
**61**	Eucalyptol **(107)**	−5.86 kcal/mol	-	-	MET49, MET165, HIS164, ARG188, HIS41, PRO52, ASP187, GLN189, TYR54, PHE18	[98]
**62**	Nictoflorin **(114)**	−9.18 kcal/mol		THR190, GLY143	-	[99]
**63**	Aloenin **(115)**	−9.13 kcal/mol		PHE140	-	[99]
**64**	Andrographolide **(116)**	−7.06 kcal/mol	-	PHE140, SER144	-	[100]
**65**	Galangin **(117)**	−8.066 kcal/mol	-	**CYS145**, PHE140, HIS164, **HIS41**	HIS41, MET165, MET49, GLU166	[101]
**65**	Amentoflavone **(42)**	−9.9 kcal/mol	-	HIS163, THR26, GLU166	GLN189, ARG188, ASP187, TYR54, MET49, HIS164, HIS172, PHE140, SER144, LEU141, GLY143, LEU27, THR25, THR26, ASN142	[102]
**67**	Kazinol T **(123)**	−14.355008 kcal/mol	-	**HIS41**	GLY143, THR190, GLY143, HIS42, **CYS145**, GLY143, HIS164	[103]
**68**	Desacetyl gedunin **(125)**	−7.3 kcal/mol	-	TYR 207, SER245, MET206, ARG166, VAL202, SER170, GLU203, LEU199, THR197, LEUA85, LYS232, MET208		[104]
**69**	Palmatine **(126)**	−8.7 kcal/mol	−71.47 kJ/mol	GLN189	ASN142, GLY143, THR25, LEU27, ARG188,GLN192, PRO168, **CYS145**, MET165, GLN189, **HIS41**	[105]
**70**	Sauchinone **(127)**	−8.9 kcal/mol	−71.68 kJ/mol	MET49, **HIS41**	ARG188, ASP187, PRO52, CYS44, **HIS41**, THR25, LEU141, GLU166, GLN189, CYS145, MET165	[105]
**71**	Quercetagetin 7-glucoside **(130)**	−15.20 kcal/mol	-	CYS44, LEU141, **CYS145**, GLU166, GLN189, **HIS41**, CYS44	LEU27, CYS44, MET49, PHE140, LEU141, **CYS145**, MET165, LEU167, PRO168	[106]
**72**	Berberine **(134)**	−7.3 kcal/mol	-	THR25, SER46	HIS163	[107]
**73**	Withanoside V **(43)**	−10.32 kcal/mol	-	ASN84, ARG40, MET 82	CYS85, ARG105, PHE134	[108]
**74**	Lignoid **(140)**	−9.0 kcal/mol	-	THR25, **HIS41**, LEU141, SER144, **CYS145**, HIS163, GLN189	LEU27, MET49, HIS163, MET165, ASP187, GLN189	[109]
**75**	Lycorine **(141)**	−11.9 kcal/mol	-	GLU166, **HIS41**	ASP188, GLN192, MET 165, GLN 189, HIS 164, HIS163, PHE 140, LEU 141, CYS 146, SER 144, ARG 188	[110]
**76**	Hemanthamine **(142)**	−11.4 kcal/mol	-	MET165, HIS163, **HIS41**	**CYS145**, PHE140, ASN142, SER144, LEU141, ARG188, HIS164, GLU166, ASP187, GLN189	[110]
**77**	NSC36398 **(143)**	−8.1 kcal/mol	-	PHE140, LEU141, SER144, MET165, GLU166, GLN189	LEU27, **HIS41**, LEU50, PHE140, LEU141, GLY143, SER144, **CYS145**, HIS163, HIS164 MET165, GLU166, LEU167, PRO168, GLN189	[111]
**78**	Quercetin 7-*O*-glucoside-3-*O*-rutinoside **(147)**	−9.47 kcal/mol	-	PRO168, GLU166, **CYS145**, MET165, MET49, GLN189	MET165, GLU166, GLN189, ASN142, MET49, PRO168, **HIS41**	[112]
**79**	Pyranonigrin A **(151)**	−7.3 kcal/mol	-	LEU141, GLY143, SER144, **CYS145**, HIS163, GLU166 GLN189, ASN142	MET165	[113]
**80**	Arboreol **(153)**	−8.2 kcal/mol	-	-	-	[114]
**81**	Acacetin **(154)**	−7.77 kcal/mol	-	MET49, TYR54, **CYS145**	MET49	[115]
**82**	Epoxy-linalool oxide **(155)**	−12.80 kcal/mol	-	LEU141, **CYS145**, GLY143	MET165, ASN142, HIS163, **HIS41**, GLU166, HIS164, SER144, LEU27, THR25, MET49, THR26	[116]
**83**	Glycyrrhizin **(156)**	−8.7 kcal/mol	-	ASP289, ASN238	TYR239, LYS137, ASP197, THR198, GLU290, SER284, TYR237, LEU287, LEU286, LEU272, GLY275, LEU271, GLN273, ASN274, MET276, TYR239	[117]
**84**	Rutin **(29)**	-	−40.293 kcal/mol	ASN142, MET49, **HIS41**, ASP187		[118]
**85**	Vobtusine lactone **(157)**	−8.3 kcal/mol	-	ASN119, SER46, THR25	CYS44, **HIS41**, MET49	[119]
**86**	Sotetsuflavone **(159)**	−9.6 kcal/mol	-	GLU166, GLN189, THR25	PRO168, LEU167, GLY170, MET165, ASP187, ARG188, TYR54, THR45, THR24, ASN142	[120]
**87**	Kaempferol **(23)**	−7.8 kcal/mol	-	SER144, LEU141, ASP187, TYR54, GLN189	HIS163, HIS164, **CYS145**, GLU166, MET165, MET49, **HIS41**, ARG188	[121]
**88**	Echoside A **(168)**	−8.4 kcal/mol	-	THR25, **HIS41**, **CYS145**, GLU 166	MET165, MET49	[122]
**89**	Echoside B **(169)**	−9.4 kcal/mol	-	ARG4, SER 284, and LYS5	LYS5, GLU288, LEU282	[122]
**90**	Juniper camphor **(172)**	−6.06 kcal/mol	-	MET165	MET49, MET165, PRO168, ASP147	[123]
**Papain-Like Protease**						
**91**	Amentoflavone **(42)**	−10.8 kcal/mol	-	HIS342, LYS711, ARG712	LYS711, ASP339, ARG558, ILE310, ILE580, ALA579, LEU742	[79]
**92**	Jezonofol **(167)**	−9.0 kcal/mol	−60.7 kcal/mol	ARG166, GLU16, GLN174, TYR264	SER170. GLY163, MET208, GLN203, LYS157, VAL202, MET206,LEU199, TYR207, LEU185, GLU161, LEU162, GLN269, TYR268	[91]
**93**	Constanolactone B **(80)**	-	−92.57 kJ/mol	TYR268, ASP164	ASN109, TYR11, GLY271, LEY162, CYS270, GLY163, GLN269, THR301, ARG166, PRO248, MET208, PRO247, ALA246, SER245	[88]
**94**	Glaucogenin D **(81)**	−6.4 kcal/mol	-	**HIS272**	**TRP106**, **HIS272**	[89]
**95**	Glaucogenin A **(82)**	−6.4 kcal/mol	-	**HIS272**, **ASP286**	**TRP106**, **HIS272**, TRP270	[89]
**96**	(*E*)-7-(4-hydroxy-3-methoxyphenyl)-1-phenylhept-4-en-3-one **(84)**	-	−47 kJ/mol (closed conformation) −28 kJ/mol (open conformation)	ARG166, ASP164, GLN269, TYR264, LEU163, GLY163, MET208 -		[124]
**97**	8-gingerol **(85)**		−43 kJ/mol (closed conformation) −15 kJ/mol (open conformation)	ARG166, ASP164, TYR268, GLN269, TYR264, PRO247, GLY163, PRO248 -		[124]
**98**	4,8-dihydroxysesamin **(152)**	−10.3 kcal/mol	-	-	-	[114]
**99**	Glycyrrhizin **(156)**	−7.9 kcal/mol	-	ASN128, ASP179, GLN174	ASP76, ASN156, ARG82, THR74, PHE79, TYR154, HIS175, HIS73, PHE69, VAL202, PHE173, LEU178, ALA176, ASN177	[117]
**100**	Rutin **(29)**	-	−21.713 kcal/mol	TYR268, GLN269		[118]
**101**	Deoxyvobtusine lactone **(158)**	−10.8 kcal/mol	-	ARG558, ARG712	ILE310, PHE735, LEU742, ASP339 THR583	[119]
**102**	6-demethoxy-4′-O-capillarsine **(170)**	−18.86 kcal/mol	-	GLN270, PRO249	TYR269, ASP165, PRO248, PRO249	[125]
**103**	Tenuflorin C **(171)**	−18.37 kcal/mol	-	GLN270, TYR274, ALA247, LEU163	TYR269, ASP165, PRO248, ASP165, MET209	[125]
**Spike Proteins**						
**104**	Terpene NPACT01552 **(173)**	−11 kcal/mol	-	GLY496, TYR453, GLN493, SER494, GLU484	LYS417, LEU452, TYR489, PHE456, LEU455	[126]
**105**	Hinokiflavone **(175)**	−9.60 kcal/mol	-	GLN1201, GLN926, GLU1195, LEU1197, ASN928, GLU918, ASN919	GLU1195, LEU1197, GLU918	[127]
**106**	Robustaflavone **(176)**	−9.40 kcal/mol	-	ASP936, GLN926, LYS1191, GLU1195, ASN928	GLU1195, ASN928	[127]
**107**	Abietatriene **(178)**	−9.8 ± 0.02 kcal/mol	-	-	TYR453, TYR495, ASN501, TYR505	[128]
**108**	Quercetin **(40)**	−7.8 kcal/mol	-	ASN501, TYR505, GLY496	GLN493, LYS417, GLU406, LEU455, TYR495, PHE497, GLN506	[77]
**109**	Hesperidin **(51)**	-	−10.4 kcal/mol	ASN1023, SER1030, LHR1027	ARG1039, ALA1026, LEU1024, PHE1042, ARG1039, THR1027, ARG1039, LEU1024, THR1027, ALA1020, ASN1023, GLN784, GLU780	[86]
**110**	Nabiximols **(179)**	-	−10.2 kcal/mol	GLN762, LYS776, GLU1017	ARG765, ARG1014, ALA958, ALA766, LEU1012, GLY769	[86]
**111**	Amentoflavone **(42)**	−9.9 kcal/mol	-	LEU861, LYS733, SER730, THR732	ALA956, PRO862, HIS1058, ASP867, VAL860, PRO1057, MET731, VAL952, ASN955, PHE823	[78]
**112**	Crocin **(63)**	−7.6 kcal/mol	-	ARG346, PHE347, SER349, TYR351, LEU441, LYS444, VAL445, GLY447, ASN448, TYR449, ASN450, PHE490		[73]
**113**	Pelargonidin-3-galactoside **(180)**	−8.6 kcal/mol	-	GLN166, PHE83	SER80, LYS103, PRO40, ASP165	[90]
**114**	Orientin **(181)**	−6.2 kcal/mol	-	LYS333, ASP429, THR431, ASN435, ASN437, TYR438, ARG495	ASN437	[129]
**115**	Chrysoeriol **(182)**	−11.478 kcal/mol	-	CYS336, GLY339, ASP364	PHE338, PHE342, PHE374, LEU335, VAL367, SER373	[130]
**116**	Luteolin **(183)**	−11.392 kcal/mol	-	ASP364, VAL367, SER371, SER373, CYS336, VAL362	PHE338, GLY339, PHE374, PHE342	[130]
**117**	Acacetin **(154)**	−7.75 kcal/mol	-	THR26, THR24, HIS164, GLU166, LEU141, SER144, CYS145, GLT143	HIS41	[115]
**118**	Epigallocatechin gallate **(21)**	−6.3 kcal/mol	-	TYR1110, PHE1109, GLN1071, GLU918, THR716	-	[131]
**119**	Geraniin **(177)**	−8.1 kcal/mol	-	ARG403, TYR449, TYR453, GLN493, SER494, GLN498	TYR495, GLY496	[132]
**120**	*cis*-Annonacin **(192)**	−7.7 kcal/mol	-	PHE390, ARG393, GLN409, GLY496, TYR505, ARG403, TYR453	ASN33, GLU37, PRO389, ARG403, GLU406, LYS417, TYR495, PHE497	[133]
**121**	Trigoneoside IB **(195)**	−8.5 kcal/mol	-	THR260, ALA618, SER289, LYS946, SER279, THR261, THR616, GLY288, THR302, THE285, CYS288	-	[134]
**122**	Arctiin **(196)**	137.043 (LiDock score)	-	SER142	THR193, ALA146, GLY144, SER196, GLY143, PRO195, PHE118, THR141, VAL194	[135]
**123**	Linarin **(197)**	162.676 (LiDock score)	-	GLY42, VAL105, GLN168	ARG44, GLY103, GLN102, TYR88, GLN43, GLN39, ALA85, GLU107, PRO41, SER170, GLU167, THR104, GLY103	[135]
**124**	Withanolide D **(198)**	−9.8 kcal/mol	-	GLN954	ASP950, VAL951, LYS947, PRO728, GLU1017, GLU773, ILE770, LEU1012, ILE1013, GLN1010, AGR1014, ALA766, GLY769	[136]
**125**	Morellic acid **(199)**	−10.3 kcal/mol	-	THR549, THR547	MET740, GLY744, ASN856, PHE855, PHE541, ILE587, THR573, LEU546, ASP571, LEU977, ARG1000, SER975, ASN978, ASN540, ASP745, GLY548	[120]
**126**	Echoside A **(168)**	−7.9 kcal/mol	-	GLU1195, LEU1200, GLN1201, ASP1199	LEU1197	[122]
**127**	Echoside B **(169)**	−7.8 kcal/mol	-	SER943, ASP936, ARG1185	LYS1191, ALA1190	[122]
**128**	Pyrrolidinone **(200)**	−5.97 kcal/mol	-	SER730	THR778, PHE782, VAL729, ILE870, ALA1056, AND GLY1059	[137]
**129**	Scutellarein **(118)**	−8.9 kcal/mol	-	-	-	[114]
**(a) RBD-ACE2**						
**130**	3-*p*-coumaroylquinic acid **(174)**	−8.9 kcal/mol	−6.71 kcal/mol	THR1006, GLN1005	ALA766, LEU763, VAL1008, GLN1010, GLN1002, THR1009, THR1006	[138]
**131**	Rutin **(29)**	−7.601 kcal/mol	-	ASN388, ASP389, ALA363, CYS361, SER359, ILE332, ASN331	-	[69]
**132**	Catechin **(41)**	−6.470 kcal/mol	-	SER359, ASN331, CYS 361, ILE332	-	[69]
**133**	Kaempferol **(23)**	−6.743 kcal/mol	-	ILE358, ASN388	-	[69]
**134**	Azadirachtin H **(184)**	−8.18 kcal/mol	-	ARG393, ARG408, LYS417, GLN409, ASP30, ASN33, HIS34, LYS31, ASP30, GLU406, LYS455, ARG403		[139]
**135**	Indigo blue **(186)**	−11.2 kcal/mol	-	GLN 947, GLN 744	PHE 741, THR 943	[140]
**136**	Echoside A **(168)**	−7.5 kcal/mol	-	ARG393, PHE390, ASN394	ALA99, LEU100, LEU73, LEU391, PHE40	[122]
**137**	Echoside B **(169)**	−8.2 kcal/mol	-	SER47	TRP349	[122]
**(b) ACE2**						
**138**	Geraniin **(177)**	−7.0 kcal/mol	-	ARG403, TYR449, TYR453, GLN493, SER494, GLN498	TYR495, GLY496	[132]
**139**	Tuftsin **(193)**	−6.9 kcal/mol	-	SER47	ASN51, HIS345, ASP67	[141]
**140**	Epoxy-linalool oxide **(155)**	−13.13 kcal/mol	-	GLN101, ASN103	GLN81, GLN98, LEU85, HIS195, ASN194, ALA193, AND GLN102	[116]
**141**	Withanolide D **(198)**	−8.1 kcal/mol	-	-	GLY352, ARG393, TRP349, ALA348, THR347, GLU402, HIS378, HIS401, ASP382, ASP350, PHE40	[136]
**142**	Morellic acid **(199)**	−12.1 kcal/mol	-	THR371, ASP368, LYS363, ASP367	CYS361, MET360, ASN149, TRP271, ASP269, THR276, THR445, HIS374, GLU406, GLU375, HIS505, TYR515	[120]
**143**	Pyrrolidinone **(200)**	−5.24 kcal/mol	-	THR434	ILE291, PRO415, PHE438	[137]
**(c) GRP78**						
**144**	Orientin **(181)**	−7.2 kcal/mol	-	MET433, LYS435, ARG439, GLU469	LYS435, PRO438, PRO471, LYS556	[129]
**(d) NRP1**						
**145**	Tuftsin **(193)**	−8.1 kcal/mol	-	ASN544	GLU550, LYS397, PRO398, LEU551	[141]
**(e) TMPRSS2**						
**146**	Withanolide D **(198)**	−9.7 kcal/mol (TMPRSS2)	-	ARG257, ARG241, TYR238	GLU253, THR396, TYR250, ASN249, THR246, GLU243, MET239, TYR250	[136]
**RNA-dependent RNA polymerase**						
**147**	α-hederin **(201)**	−8.6 kcal/mol	-	ASP760, CYS622, ARG553	THR556, ALA688, LYS500, ASP623, SER682	[142]
**148**	Kaempferol **(23)**	−7.0 kcal/mol	-	MET87	ASN414, ASN416, GLN18, GLN19, SER15, ASP846, LYS411, PRQ412, TYR546	[90]
**149**	Ellagitannin punicalin **(202)**	−9.5 kcal/mol	-	ASN497, GLY590, ASP684, TYR689	ILE494, LYS577, ASP684, ALA685	[79]
**150**	Cyanidin **(203)**	−7.7 kcal/mol	-	**ASP761**, TRP617, TRP800, GLU811	CYS622, TYR619, **ASP760**, ASP618, ALA762, GLY616, PHE812	[77]
**151**	Amphimedoside C **(78)**	-	−47.9 kJ/mol	ASP623, LYS621, ALA554, ASP452, ARG553	ARG553	[87]
**152**	14-debromoaraplysilin I **(204)**	-	−111.52 kJ/mol	ALA762, ASP623, ARG555, ARG553	TRP617, CYS622, PHE694, VAL764, GLY616, VAL763, ASN695, ASP618, TYR619, **ASP760**	[88]
**153**	Glaucogenin D **(81)**	−7.3 kcal/mol	-	TRP619, **ASP760**	-	[89]
**154**	Glaucogenin C **(205)**	−7.3 kcal/mol	-	GLU811, **ASP760**	LYS798	[89]
**155**	Crocin **(63)**	−10.5 kcal/mol	-	ASN497, ASP499, LYS500, LYS545, ILE548, ARG836, ASP845, ARG858		[73]
**156**	Jezonofol **(167)**	−10.5 kcal/mol	−30.7 kcal/mol	ARG836, A19, A11, U9, LEU544, ASP845, U18, U17	ALA574, ILE548, ILE847, TYE546, LYS545, VAL557, U20	[91]
**157**	Ellagic acid **(206)**	−6.4 kcal/mol	-	GLY808, THR817, PRO809, HIS816, TYR831	HIS810, LYS807, GLU802,	[121]
**158**	Vobtusine lactone **(157)**	−8.7 kcal/mol	-	SER682, ALA688	THR687, ASP684, ASN497, ALA685, TYR689, ARG569, GLN573, ILE494, LEU576, LYS577, ALA580, **SER759**, GLY590	[119]
**159**	Echoside A **(168)**	−7.3 kcal/mol	-	ASN781, SER709, LYS47	LYS780, GLY774, ALA706	[122]
**160**	Echoside B **(169)**	−8.0 kcal/mol	-	LYS47, SER709, LYS714, THR710	LYS 780	[122]
**161**	Arboreol **(153)**	−8.9 kcal/mol	-	-	-	[114]
**Nucleocapsid**						
**162**	4,8-dihydroxysesamin **(152)**	−10.7 kcal/mol	-	PRO163, THR166, LEU162, GLY70	GLU137, GLY165, GLN164, THR167, THR77, ASN76, GLN84, SER79, PRO81, PRO163, THR136	[114]
**163**	Arboreol **(153)**	−10.6 kcal/mol	-	GLN84, PRO163, GLY70	ILE75, GLN164, GLY165, THR166, GLU137, LEU162, PRO81, PRO163, THR136	[114]
**164**	Glycyrrhizin **(156)**	−7.9 kcal/mol	−30.05 kcal/mol	THR92, ARG94, ARG89, TYR110, ARG150	ARG90, ALA91, ASN49, THR50, ALS51, PHE54, THR55, TYR112, PRO118, PRO152, ALA157	[117]
**165**	Rutin **(29)**		−34.342 kcal/mol	-	-	[118]
**166**	Trigoneoside IB **(195)**	−7.6 kcal/mol	-	ARG71, ARG70, THR60, SER29, ALA28, THR27, GLY125, ILE124, TYR90	-	[134]
**167**	Apigenin **(120)**	−8.11 kcal/mol	-	PHE67, PRO68, ARG69, GLY70, GLN71, TYR124, TRP133, VAL134, THR136, GLY138, ALA139		[143]
**168**	Curcumin **(86)**	−8.75 kcal/mol	-	LEU161, GLN163, ALA173		[144]
**Endoribonuclease**						
**169**	Hinokiflavone **(175)**	−8.6 kcal/mol	-	MET243	MET243, TYR262, GLU258, HIS362, ALA256	[79]
**170**	Rutin **(29)**	−8.68 kcal/mol	-	LEU246, GLY248, GLN245, CYS291, **THR341**, **HIS250**	LYS345, LEU346, TYR343	[85]
**171**	Glucopyranoside derivative **(211)**	-	−263.640 kJ/mol	GLU340, GLN245, **HIS235**, LYS290, GLY248, VAL292, SER294	TRP333 MET331 LYS345, PRO344, VAL318, TYR343, GLN347, GLY247, HIS243, ASP240, LEU246, **THR341**, **HIS250**, CYS293	[145]
**172**	4-((2S,3R)-3-(hydroxymethyl)-5-((E)-3-hydroxyprop-1-en-1-yl)-7-methoxy-2,3-dihydrobenzofuran-2-yl)-2-methoxyphenol **(212)**	−6.26 kcal/mol	−87.52 kcal/mol	-	-	[146]
**Helicase**						
**173**	Crocin **(63)**	−9.5 kcal/mol	-	ALA18, ILE20, CYS112, ASP113, TRP114, THR141, PHE145, GLY415, HIS482, ASP483, VAL484, SER485, TYR515, THR552, HIS554		[73]
**174**	Rhusflavanone **(213)**	−9.2 kcal/mol	-	GLU341, ASP534	ALA312, ALA313, VAL340	[79]
**175**	Morelloflavone **(214)**	−9.2 kcal/mol	-	LYS288, ALA316, ARG443	THR286, ALA316, LYS320, GLY538, SER539	[79]
**176**	Chromone **(215)**	−6.24 kcal/mol	−90.99 kcal/mol	-	-	[146]
**177**	Chromone **(216)**	−6.24 kcal/mol	−90.99 kcal/mol	-	-	[146]
**178**	Scirpusin A **(217)**	−8.9 kcal/mol	−41.9 kcal/mol	PRO514, ASN516	ARG560, SP534, ASN177, GLU201, LYS202	[91]
**Methyl transferase (NSP16–NSP10 complex)**						
**179**	Robustaflavone **(176)**	−10.6 kcal/mol (NSP16) −7.7 kcal/mol (NSP10)	- -	ASP6897, ASP6928 ASP4335	CYS6913, CYS6914, MET6929, ASP6931, PHE6947, GLY6869, LEU6898 ARG4331, ILE4334, LYS4346	[79] [79]
**180**	Michellamine B **(218)**	−10.6 kcal/mol (NSP16)	-	LYS6844, CYS6913, ASP6928, ASP6928, ASN6996	ASN6841, ASP6897, GLY6869, MET6929, LEU6898, GLU7001	[79]
**181**	Chromone **(219)**	−6.20 kcal/mol	−75.24 kcal/mol	-	-	[146]
**ADP phosphatase/ADPRP**						
**182**	Crocin **(63)**	−8.2 kcal/mol	-	ASP22, LYS44, GLY48, ALA154, PHE156		[73]
**Exoribonuclease**						
**183**	Chromone **(220)**	−7.09 kcal/mol	−81.16 kcal/mol	-	-	[146]
**Other Nonstructural proteins**						
**184**	Hispaglabridin B **(221)**	−8.5 kcal/mol	−42.88 kcal/mol	AASP61, GLY62, THR63, LEU45, ARG11, LEU10	ALA55, ARG100, VAL103, ILE66, VAL42	[147]
**185**	Licoflavone B **(222)**	−8.1 kcal/mol	−42.76 kcal/mol	MET13, GLY62	GLY64, ILE66, LYS93, LEU95, ALA31, ARG11	[147]
**186**	Ochnaflavone **(223)**	−9.1 kcal/mol	−41.43 kcal/mol	GLY94, ARG40, **ASP301**	THR68, ILE92, GLY39, GLY38, PHE57, LYS59, MET13, LYS93, LEU95, SER60, PHE41, VAL42, ILE66, ARG40, **GLU331**	[147]
**187**	2,3-dehydrosomnifericin **(224)**	−12.3 kcal/mol	-	LEU126	PHE132, ILE131, VAL49	[148]
**Envelope proteins**						
**188**	Trigoneoside IB **(195)**	−7.5 kcal/mol	-	ILE124, TYR90, ARG70, THR60, SER29, ALA28, THR27, GLY125	-	[134]
**189**	Dicumarol **(225)**	−7.4 kcal/mol	-	-	-	[114]

* The catalytic residues are shown in blue, and the binding site residues are shown in black.

## Data Availability

Not applicable.
